# The carbon footprint of Chimeric Antigen Receptor-T cell manufacturing processes

**DOI:** 10.1016/j.ijpx.2026.100654

**Published:** 2026-08-28

**Authors:** Wietske E. van der Bijl, Bahez Gareb, Koen M. Klomberg, Edwin Bremer, Tom van Meerten, Thijs H. Oude Munnink, Bart G.J. Dekkers

**Affiliations:** a Department of Clinical Pharmacy and Pharmacology, University of Groningen, University Medical Center Groningen, Groningen, the Netherlands; b Department of Hematology, University of Groningen, University Medical Center Groningen, Groningen, the Netherlands.

**Keywords:** Chimeric Antigen Receptor T (CAR-T) cell therapy, Carbon footprint, Life cycle assessment

## Abstract

Chimeric Antigen Receptor T (CAR-T) cell therapy is an established treatment for haematological malignancies, yet the environmental impact of its manufacturing and administration remains unexplored. As healthcare systems aim to reduce their carbon footprint, understanding the climate impact of CAR-T cell manufacturing is essential. The objective of this study was to quantify the carbon footprint, expressed in CO_2_-equivalents (CO_2_e), of CAR-T cell manufacturing and identify process steps that contribute most to CO_2_-emissions. A systematic life cycle assessment (LCA) comparing centralized and point-of-care (PoC) CAR-T cell manufacturing processes was conducted, according to ISO14044:2006 standards. All major processes were assessed, including leukapheresis, cryopreservation, transport, manufacturing, background facilities, quality controls, and infusion. Emissions were calculated using openLCA and open-access databases. Transport was the dominant contributor to total emissions, accounting for the higher footprint of centralized manufactured CAR-T cells (355.75 kg CO_2_e) compared to PoC CAR-T cells (72.85 kg CO_2_e). In addition, open system background facilities emitted substantially more CO_2_ compared to closed system background facilities (85.16 vs. 25.00 kg CO_2_e). Reducing travel distance and optimizing cleanroom use offer the greatest potential for emission reduction. This study provides the first assessment of differences in the carbon footprint of two established CAR-T cell manufacturing platforms and offers a framework that can be applied across a broad range of CAR-T products. It highlights transport and cleanroom energy use as key drivers of emissions, causing PoC manufacturing to substantially reduce the environmental impact by limiting long-distance transport.

## Highlights

• The first carbon footprint assessment of CAR-T cell manufacturing processes.
• Transport drives CO_2_ emissions differences between point-of-care and centralized CAR-T cell manufacturing.
• Transport emissions cause point-of-care manufacturing to be more sustainable.
• Optimizing cleanroom efficiency and reducing transport can reduce emissions.

## Graphical abstract

**Figure:**
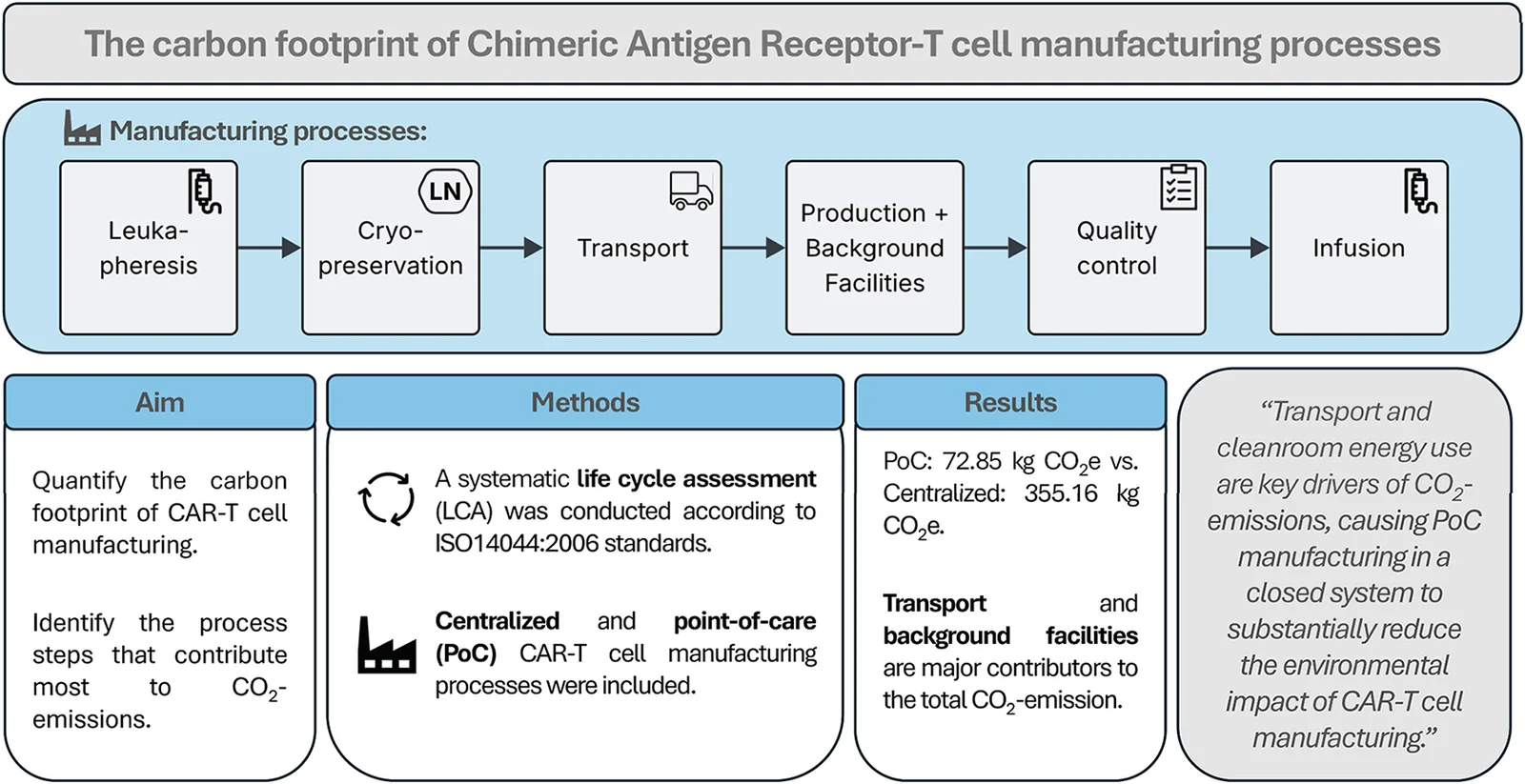

## 1 Introduction

Chimeric Antigen Receptor T (CAR-T) cell therapy is a form of immunotherapy that has changed the treatment landscape for haematological malignancies (Zhang et al., 2026), and is also under investigation for the management of solid malignancies. The manufacturing of CAR-T cells is complex and patient-specific, requiring autologous T cell collection, genetic modification, expansion, and extensive quality control testing.

Commercially available CAR-T products, such as axicabtagene ciloleucel (axi-cel, Yescarta®), are manufactured in centralized facilities. This manufacturing platform involves cryopreservation of the starting material (leukapheresis material) and final CAR-T cell product, as well as transport between hospital and manufacturing site. As a result, the manufacturing process of commercially available CAR-T products ranges from four to six weeks (Locke et al., 2025).

CAR-T cells manufactured as point-of-care (PoC), such as ARI-0001 or CAR19, offer a substantially shorter manufacturing time of one to two weeks, potentially leading to a clinically relevant improvement for patients with rapidly progressing disease (Castella et al., 2020; Maschan et al., 2021; Ortíz-Maldonado et al., 2021). The product can be formulated as a suspension containing fresh cells. Alternatively, the product can also be cryopreserved when clinically indicated, such as in cases of treatment postponement.

On top of clinical advantages, PoC manufacturing may also reduce the environmental impact of CAR-T cell manufacturing. Globally, healthcare accounts for approximately 4.9% of total carbon emissions (Keil et al., 2024). In the Netherlands, this percentage is estimated at 7% (Steenmeijer et al., 2022). To address this impact, the Dutch Green Deal Sustainable Healthcare was established, which includes the reduction of the environmental impact of medication as one of its key themes (“Green Deal Duurzame Zorg,” 2022).

Insight into the carbon emission associated with different manufacturing processes is currently limited, complicating sustainable decision-making in the context of sustainability. Differences in manufacturing processes are likely to influence the carbon footprint of CAR-T cell manufacturing.

In this study, we aimed to perform a systematic life cycle assessment (LCA) of CAR-T cell manufacturing processes. The results of this study are not intended to guide decisions on whether or not to use PoC CAR-T manufacturing, since this decision is highly dependent on many other factors and national frameworks. Rather than focusing solely on end-product comparisons, this study compares variations within manufacturing processes and their contributions to the overall carbon footprint, expressed in CO_2_-equivalents (CO_2_e). By doing so, this study aims to identify the manufacturing protocol with the lowest environmental impact and identify options for the reduction of carbon dioxide emissions. Ultimately, the aim is to reduce the environmental impact of CAR-T cell manufacturing.

## 2 Materials and Methods

A systematic LCA was conducted to quantify the carbon footprint of CAR-T cell manufacturing, expressed as CO_2_-equivalents. The methodological framework follows the ISO14044:2006 international standard (including Amendments 1:2017 and 2:2020) (International Organisation for Standardisation (ISO), 2006), and incorporates pharmaceutical product-specific recommendations (Siegert et al., 2019). The LCA was performed in OpenLCA using open-access databases, such as carboncloud (Carboncloud, 2026; OpenLCA, 2025). ReCiPe 2016 Midpoint (H) was used for the calculations in OpenLCA. In addition, information on energy consumption of different systems was retrieved from the available information, such as user manuals and technical data forms. For some systems, such as the CliniMACS Prodigy, the manufacturer was contacted to obtain additional information on energy consumption. When there was insufficient data available on energy consumption, assumptions were made as described in the Supplementary data (including missing inventory data).

### 2.1 Goal and scope definition

**Fig. 1:**
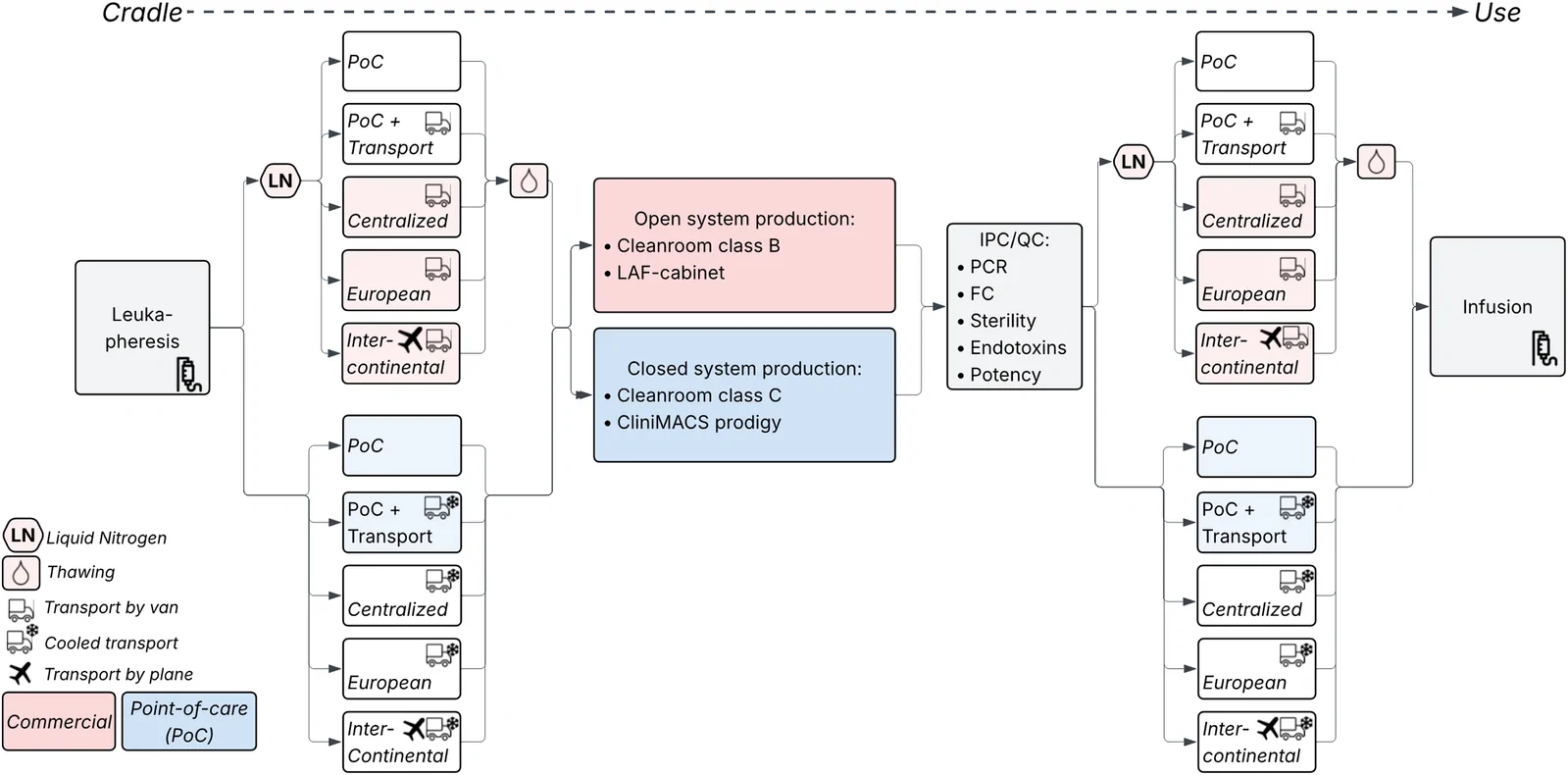
Graphical overview and system boundaries of the life cycle assessment of the different processes involved in the manufacturing of CAR-T cells.

A cradle-to-use life cycle model for CAR-T cell manufacturing was developed to assess the impacts from leukapheresis to infusion (Fig. 1). All relevant steps in the manufacturing process and administration were incorporated, including leukapheresis, cryopreservation, transport, CAR-T manufacturing, background facility, quality control, and infusion. The assessed impact category is the global warming potential, expressed as kilograms of CO_2_-equivaltents (kg CO_2_e) per functional unit and the functional unit is defined as the manufacturing and infusion of a single autologous CAR-T cell product for one patient. For the centrally manufactured product this is defined as a cryoproduct, whereas for the PoC product this concerns a fresh product. Additional methodological specifications on the LCA are provided in Supplementary data chapter 1.

### 2.2 Life cycle inventory analysis

#### 2.2.1 Leukapheresis

The first step in the manufacturing of CAR-T cells is leukapheresis. The materials required for this process are listed in Supplementary data chapter 2.1. Only the leukapheresis procedure itself is included in this study. Preparatory steps, such as pre-medication and patient transport, are excluded.

#### 2.2.2 Cryopreservation

Following apheresis, T cells are either cryopreserved or transported directly to the manufacturing facility. With cryopreservation, this typically occurs both before and after manufacturing. In general, centrally manufactured CAR-T cells are cryopreserved, whereas PoC CAR-T cells are used fresh. The materials needed for cryopreservation are provided in Supplementary data chapter 2.2. The freezing procedure, storage in a cryogenic freezer, liquid nitrogen used for transport and thawing process are all included in the LCA. In contrast, the back-up cryopreserved products and samples for quality control are excluded, as these are consistent across both manufacturing methods and therefore expected to contribute to differences in CO_2_ emissions.

#### 2.2.3 Transport

For transport, this study distinguishes five scenarios: PoC, PoC with transport, centralized manufacturing with national transport, centralized manufacturing with transport within Europe and centralized manufacturing with intercontinental transport. PoC refers to on-site manufacturing without transport. PoC with transport involves shipment from a Dutch academic hospital to the UMCG and vice versa after manufacturing. Centralized manufacturing with national transport refers to shipment from a Dutch hospital to a centralized, commercial CAR-T cell manufacturer in the Netherlands and vice versa. European transport is defined as shipment from a centralized, commercial CAR-T cell manufacturer in the Netherlands to LMU Klinikum in Munich as an average transport distance in Europe, whereas intercontinental transport refers to shipment from an academic hospital in the Netherlands to a centralized, commercial CAR-T cell manufacturer in Santa Monica (USA). When transporting non-cryopreserved products, a cooling unit is included. Transport distances and impact are estimated in Supplementary data chapter 2.3.

#### 2.2.4 CAR-T cell manufacturing

Upon arrival at the manufacturing facility, CAR-T cell manufacturing initiates. Manufacturing can be performed using either open or closed systems. The PoC product is manufactured in a closed system, which is common for PoC CAR-T cell manufacturing. A closed system involves a bioreactor, the CliniMACS Prodigy in this study, and uses a class C cleanroom as the background facility. Centrally produced CAR-T cells may be manufactured in closed and open systems, the latter of which includes CAR-T cell manufacturing in a class A LAF-cabinet with a class B cleanroom as background. To fairly compare the use of an open and closed system, this study applies a direct adaptation of the PoC manufacturing protocol to an open system. Details on materials and background facilities for the manufacturing phase are provided in Supplementary data chapters 2.4–2.6.

#### 2.2.5 In-process controls and quality control

During manufacturing, several in-process controls (IPC's) and quality controls (QC's) are performed. These include sterility testing, PCR assays, endotoxin testing, flow cytometry, and potency assays. Potency assays are either performed using a bead-based or cell-based protocol. In this report, two sterility assays, four PCR assays, two endotoxin tests, eight flow cytometry analyses, and one bead-based potency assessment are included as IPC's and QC's. More information on materials needed for these controls is provided in Supplementary data chapter 2.7.

#### 2.2.6 Infusion

Finally, after transport back to the hospital, the CAR-T product is infused into the patient. During this procedure, the CAR-T product is administered using an IV administration set, followed by flushing the line with a 0,9% NaCl bag. Additional details are provided in Supplementary data chapter 2.8.

## 3 Results

Detailed information on the carbon emissions of the relevant steps in the manufacturing process and administration (Fig. 1) can be found in the Supplementary materials.

### 3.1 Leukapheresis

**Fig. 2:**
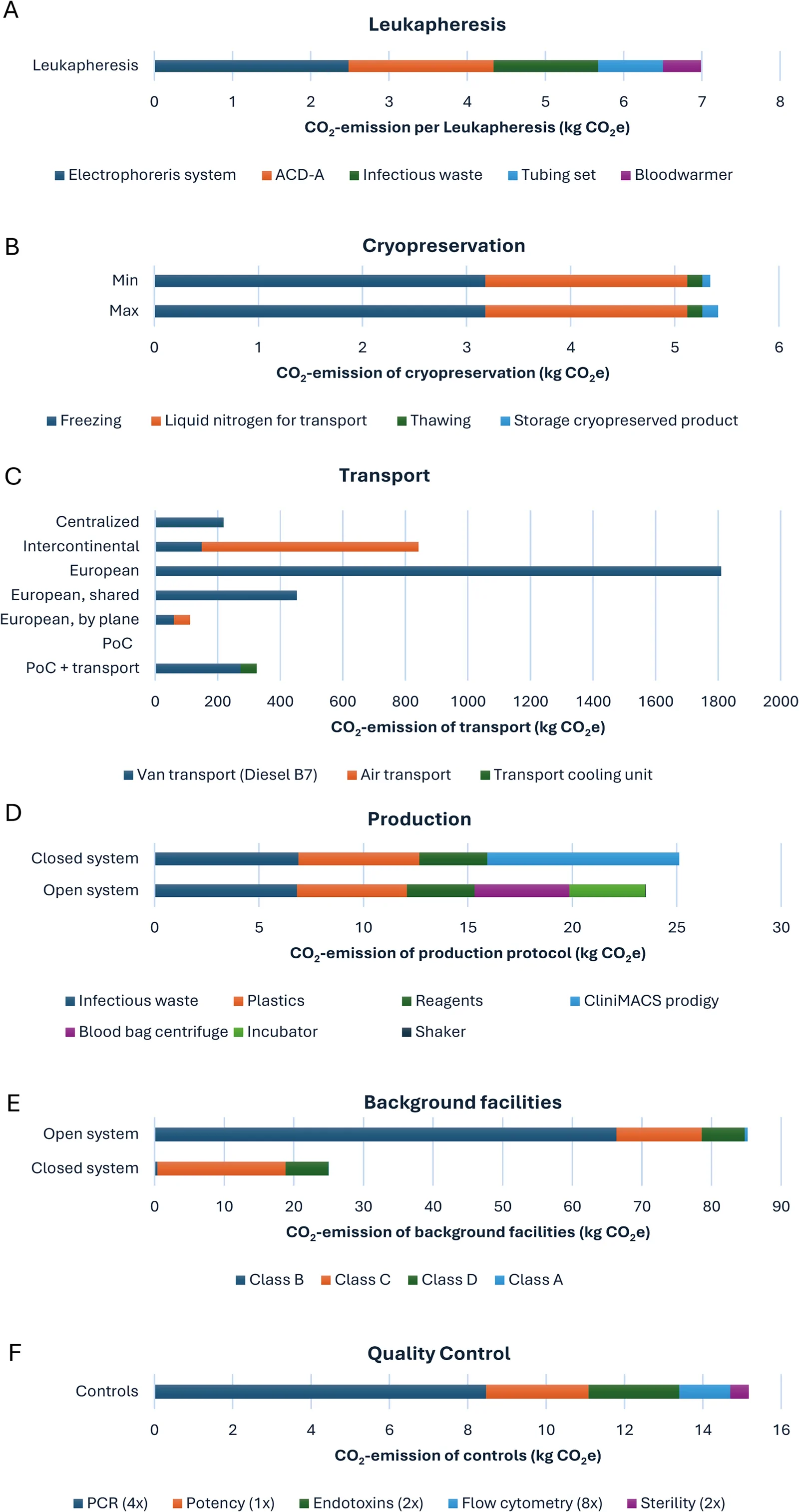
CO_2_-emission of separate CAR-T cell manufacturing processes. A) CO_2_-emission of leukapheresis in kg CO_2_e, and the contribution of the electrophoresis system, Anticoagulant Citrate Dextrose Solution A (ACDA), infectious waste, the tubing set and the blood warmer to this emission. B) CO_2_-emission of cryopreservation in kg CO_2_e, and the contribution of freezing, transport, thawing and storage to this emission. C) CO_2_-emission of transport in kg CO_2_e, and the contribution of van transport (Diesel B7), air transport and the transport cooling unit to this emission. Centralized, Intercontinental, dedicated and shared European transport, Point-of-care (PoC) and PoC + transport are compared. Central = Transport from Hoofddorp (The Netherlands) to an academic hospital in the Netherlands. Intercontinental = Transport from the USA to an academic hospital in the Netherlands. European = Transport from Hoofddorp to LMU Klinikum in Munich, Germany. PoC = Manufacturing takes place at the site where the apheresis and infusion take place. PoC + transport = Transport from the UMCG to an academic hospital in the Netherlands. Shared transport = combined transport of 4 separate units. D) CO_2_-emission of the production protocol in an open or closed system in kg CO_2_e, and the contribution of materials and electronic devices to this emission. E) CO_2_-emission of background facilities in an open or closed system in kg CO_2_e, and the contribution of each cleanroom class to this emission. F) CO_2_-emission of in process controls and quality controls in kg CO_2_e, and the contribution of each test to this emission.

The total carbon emission associated with the leukapheresis procedure is 6.99 kg CO_2_e per CAR-T cell product, with the electrophoresis system (2.48 kg CO_2_e), the Anticoagulant Citrate Dextrose Solution A (1.85 kg CO_2_e) and infectious waste (1.34 kg CO_2_e) being the main contributors in this step (Fig. 2A; Supplementary data chapter 2.1).

### 3.2 Cryopreservation

Cryopreservation is only applied when a product is transported frozen. The total CO_2_-emission associated with 21 days of liquid nitrogen storage, two freezing cycles, two liquid‑nitrogen transport steps, and two thawing procedures ranges from 5.34 to 5.42 kg CO_2_e, depending on the occupancy of the cryogenic freezer (Fig. 2B; Supplementary data chapter 2.2). The main contributors are freezing (3.18 kg CO_2_e) and liquid‑nitrogen transport (1.94 kg CO_2_e). For further calculations, the minimum value of 5.34 kg CO_2_e was used. The total CO_2_-emission of cryopreservation depends on the duration of storage and the number of IV bags stored in the cryogenic freezer and can be calculated using the equation in Supplementary data chapter 3.1.

### 3.3 Transport

Commercial, centrally manufactured CAR-T cells transport generates 218.38 kg CO_2_e for three round trips, corresponding to one round trip for the apheresis material, one for the CAR-T cell product and one for the return of transport packaging. When CAR-T cells were manufactured in the United States, intercontinental transport-related emissions increased substantially to 842.06 kg CO_2_e corresponding to two round trips by van and one round trip by airplane. Surprisingly, the emission increased even further (1809.62 kg CO_2_e) when centrally manufactured CAR-T cells were transported by dedicated transport for the product by van to a Munich as destination more central in Europe. As expected, emissions are reduced by combining transport of multiple units (452.58 kg CO_2_e for 4 units). Surprisingly, air transport within Europe also resulted in a lower CO_2_-emission. Simultaneously, during transport of PoC CAR-T cell products to other Dutch academic hospitals, the CO_2_-emission amounted to 324.31 kg CO_2_e for two round trips (Fig. 2C; Supplementary data chapter 2.3). For alternative travel distances or durations, the CO_2_-emission can be calculated using the equations in Supplementary data chapter 3.2.

### 3.4 CAR-T cell manufacturing

Manufacturing of CAR-T cells in a closed system using the CliniMACS Prodigy resulted in a total CO_2_-emission of 25.12 kg CO_2_e, whereas open system manufacturing generated 24.14 kg CO_2_e (Fig. 2D; Supplementary data chapters 2.4–2.5). In the closed system, the CliniMACS Prodigy contributed predominantly (9.19 kg CO_2_e), followed by infectious waste (6.89 kg CO_2_e) and plastics (5.79 kg CO_2_e). In contrast, within the open system, infectious waste was the major contributor (6.82 kg CO_2_e), alongside plastics (5.26 kg CO_2_e) and electronic devices, such as the blood bag centrifuge (4.54 kg CO_2_e), and the incubator (3.63 kg CO_2_e).

### 3.5 Background facility

The background facility of the open system resulted in a total CO_2_-emission of 85.16 kg CO_2_e. In comparison, the background facility of the closed system generated 25.00 kg CO_2_e (Fig. 2E; Supplementary data chapter 2.6). These values reflect a single autologous CAR-T cell manufacturing conducted in one cleanroom at a time, assuming production rooms of 15 m^2^ and personnel and material airlocks of 10 m^2^. When multiple productions are carried out within the same cleanroom, or if cleanrooms of different sizes are used, the CO_2_-emission can be calculated using the equation in Supplementary data chapter 3.3.

### 3.6 In process controls and quality controls

The quality control tests were divided into five categories, defined as sterility testing (0.23 kg CO_2_e), PCR assays (2.12 kg CO_2_e), endotoxin testing (1.16 kg CO_2_e), flow cytometry (0.16 kg CO_2_e), and potency assays. The latter can be performed using either a bead-based assay (2.62 kg CO_2_e) or a cell-based assay (2.78 kg CO_2_e). The impact of the sterility assay is primarily driven by the energy consumption of the incubator, which depends on the number of Petri dishes placed inside. The equation in Supplementary data chapter 3.4 can be used to calculate the CO_2_-emission of the sterility assay based on different numbers of Petri dishes. In the UMCG, the PoC manufacturing process includes two sterility assays, four PCR assays, two endotoxin tests, eight flow cytometry analyses, and one bead-based potency assessment, resulting in a cumulative CO_2_-emission of 15.17 kg CO_2_e (Fig. 2F; Supplementary data chapter 2.7). When alternative numbers of control assays are performed, the CO_2_-emission of the individual tests can be used to calculate the cumulative CO_2_-emission.

### 3.7 Infusion

During infusion, the IV administration set (0.17 kg CO_2_e) and the 0.9% NaCl infusion bag (0.40 kg CO_2_e) together resulted in a CO_2_-emission of 0.57 kg CO_2_e (Supplementary data chapter 2.8).

### 3.8 The impact of PoC CAR-T cells compared to centrally manufactured CAR-T cells

**Fig. 3:**
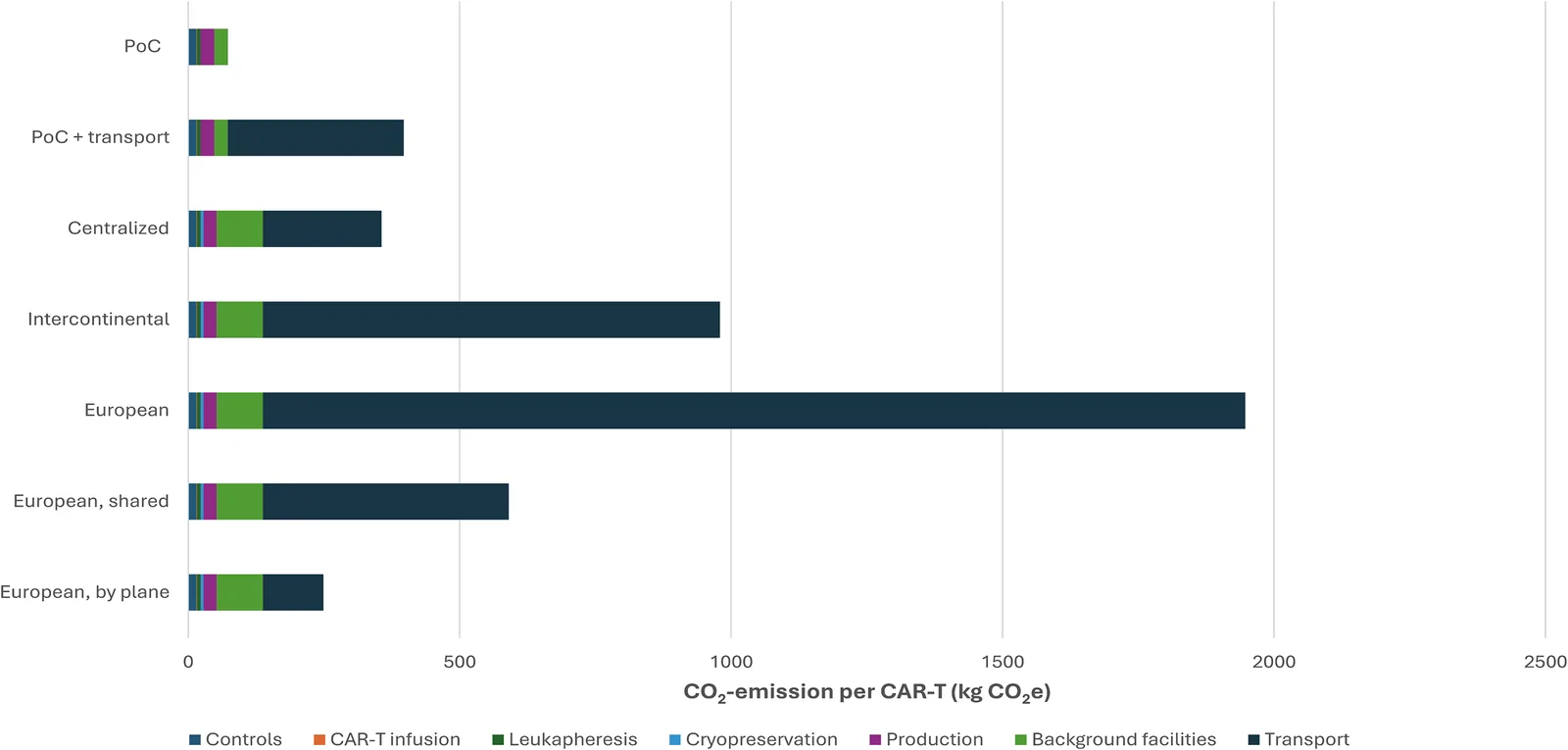
CO_2_-emission of PoC, PoC + transport, Centralized (NL), Intercontinental (USA) and Centralized European (EU) CAR-T cell products with dedicated, shared and air transport in kg CO_2_e, and the contribution of manufacturing processes leukapheresis, cryopreservation, transport, manufacturing, controls, background facilities and infusion to this emission.

The highest CO_2_-emission was observed for a closed system, centralized European CAR-T cell manufacturing (1946.99 kg CO_2_e). This value was reduced by half for intercontinental manufacturing (979.43 kg CO_2_e). PoC manufacturing with transport (397.16 kg CO_2_e) and centralized manufacturing (355.75 kg CO_2_e) resulted in lower emissions. The lowest emissions were achieved for PoC manufacturing (72.85 kg CO_2_e). The predominant contributor across scenarios is transport, while manufacturing and background facilities also contributed considerably to the total CO_2_-emission (Fig. 3).

## 4 Discussion

This study is the first to evaluate the carbon footprint associated with CAR-T cell manufacturing. The LCA quantified the CO_2_-emission of the manufacturing processes leukapheresis, cryopreservation, transport, CAR-T manufacturing, background facilities, quality control, and infusion, resulting in a clear overview of the contribution of each step to the total CO_2_-emission of CAR-T cell manufacturing. The separate reporting of process-specific emissions, combined with equations for recalculation, establishes a reproducible framework that can be applied across a broad range of CAR-T cell products and manufacturing scenarios.

The findings demonstrate that transport is the dominant contributor to CAR-T-related CO_2_-emissions. This explains why centrally manufactured CAR-T cells have a substantially higher emission than PoC products. Notably, the observed transport contribution differs from previous literature, which reports an approximate 9% contribution for transport to scope 1 and 2 of pharmaceutical companies (de Bruin et al., 2019). This discrepancy can be explained by the fact that CAR-T cell products require separate transport for each autologous product making bulk transport impossible.

It may seem counterintuitive that intercontinental transport can result in lower emissions than transport within Europe, but this can be explained by differences in type of transport. Within Europe, products are currently transported individually by dedicated road transport, whereas intercontinental transport commonly uses the cargo hold of an aircraft, allowing transport emission to be distributed across a large number of products. These emission can be reduced significantly by combining transport. Still, PoC manufacturing substantially reduces the emissions by eliminating long-distance transport, and when transport is unavoidable, minimizing travel distance or combining transport is essential.

Other relevant contributors to differences between centralized and PoC manufacturing are the manufacturing and the background facilities. While the closed and open system production protocols result in comparable emissions, the background facilities for the open system generate substantially higher emissions. This finding aligns with a previous study on the environmental impact of manufacturing advanced therapy medicinal products, confirming higher annual emissions for the controlled environments in an open system (Pinnetta et al., 2023).

From a mitigation perspective, the greatest potential for reducing emissions in the production phase lies in optimizing the use of background facilities during the manufacturing. Increasing the effective use of cleanroom floor area and decreasing the energy and material consumption through improved cleanroom design can reduce emissions per manufactured product (Partonia et al., 2024). In addition, manufacturing was performed in a class C cleanroom despite GMP requirements for ATMP manufacturing allowing the use of a class D environment (European Commission, 2017). This provides opportunities to further lower CO_2_-emissions and improve manufacturing sustainability. Conversely, manufacturing of purified CD4+ and CD8+ CAR-T products, such as lisocabtagene maraleucel, may double CO_2_-emissions during manufacturing (Abramson et al., 2020).

As the field of LCAs in healthcare is still emerging, not all relevant data is currently available. For many of the used materials, the CO_2_ emissions are currently unknown. As these materials or similar products are used in both scenario's, the impact of the missing data on the difference is likely to be limited. For the absolute footprints, the calculated emissions are expected to be at the lower bound for CAR-T manufacturing. Future studies could include direct energy metering or submetering in the analysis to more adequately determine energy consumption. Moreover, this study relied exclusively on open-access data, which is less complete than restricted-access databases. As a result, emission values for certain disposables were incomplete, particularly affecting quality controls and the manufacturing. Although this likely leads to an underestimation of absolute emissions, the comparative results remain robust because the missing materials are used in both open- and closed-system manufacturing. Similarly, incomplete information on the material composition of laboratory and medical devices may have influenced absolute values, but is unlikely to have affected the relative differences between systems. However, this could change when differences in energy composition between different vector manufacturing methods become available as contributor since this vector production is assumed to have high energy consumption.

Methodological limitations and assumptions are also inherent to LCA research. Although assumptions were kept to a minimum, some remain unavoidable. For example, PoC CAR-T cell manufacturing at the UMCG is performed exclusively in a closed system, resulting in limited knowledge of open-system manufacturing. To enable a fair comparison, this study translated the closed system protocol into an open system.

In addition to the uncertainties inherent to LCA research, this study has several limitations. First, cleanroom energy varies considerably between facilities due to differences in airflow, occupancy, leakage, cleanroom size, filtration systems, and HVAC configurations (Bunnak et al., 2016; Cleanroom Technology, 2014; The British Standards Institution, 2013). In our study, energy consumption per square meter was based on the measurements reported for monoclonal antibody manufacturing, expressed as actual device-level energy use (Bunnak et al., 2016). While this provides a realistic estimate, it should be noted that the energy consumption of individual cleanrooms may differ.

Secondly, the scope of this study was limited to the manufacturing of CAR-T cells and did not include other aspects involved in the healthcare process of CAR-T cell treatment. Although preliminary results have previously suggested that CAR-T cell therapy reduced transport related carbon emissions (Soares et al., 2025), data on other aspects of CAR-T cell therapy remain to be investigated. Data on the efficacy and safety of PoC produced CAR-T in diffuse large B-cell lymphoma is still under evaluation in the HOVON 161 trial (ClinicalTrials.gov, 2024). However, safety data on axicabtagene ciloleucel showed a high grade of cytokine-release-syndrome (CRS any grade: 86.3%; CRS grade ≥ 3: 8.2%) and Immune Effector Cell-Associated Neurotoxicity Syndrome (ICANS any grade: 47.6%; ICANS grade ≥ 3: 19.0%) (Jacobson et al., 2024). This results in the need for additional medication, such as corticosteroids, and hospitalization or intensive care. Additionally, CD28-costimulated CAR-T cells (axicabtagene ciloleucel) have been associated with higher toxicity compared with 4-1BB-costimulated CAR-T cells (tisagenlecleucel), consequently leading to higher healthcare resource utilization (Gagelmann et al., 2024). These factors may further increase the difference in environmental impact between PoC and commercial CAR-T cells. Staff-related factors, such as cleanroom clothing and travel to work, were also excluded. Transport accounts for 22% of healthcare emissions, of which 77% is accounted for by staff (de Bruin et al., 2019). Given this high contribution and the fact that open system manufacturing involves more manual handling in a higher cleanroom class, staff-related emissions are expected to cause an important increase in the total CO_2_-emission of commercial CAR-T cell products. While this study does show a considerable lower CO_2_-emission for PoC manufacturing of CAR-T cells compared to commercial manufacturing, further research should consider the whole care pathway, including both staff- and patient-related factors. Once results of the HOVON 161 trial are available, such an integrated assessment will be possible. Ultimately, this will enable to calculate the carbon footprint of a CAR-T treatment per patient, instead of per product, and this can also include the impact of failed batches.

## 5 Conclusions

This study provides the first comprehensive assessment of the carbon footprint of CAR-T cell manufacturing under the modelled assumptions and identifies the key drivers of difference in emissions between centralized and PoC manufacturing. The magnitude of the benefit, including sensitivity and uncertainty analyses should be confirmed in future studies. Transport emerged as the dominant source of emissions, highlighting the clear advantage of PoC manufacturing in reducing long-distance transport. In addition, manufacturing processes and background facilities contributed considerably to the overall footprint, underscoring the importance of optimizing cleanroom use and improving the energy efficiency of manufacturing environments. To reach the goals of reducing the environmental impact of medication (Green Deal Duurzame Zorg, 2022), integrating environmental considerations into manufacturing strategies will be essential.

## CRediT authorship contribution statement

**Wietske E. van der Bijl:** Writing – original draft, Visualization, Formal analysis, Conceptualization. **Bahez Gareb:** Writing – review & editing, Supervision, Conceptualization. **Koen M. Klomberg:** Writing – review & editing. **Edwin Bremer:** Writing – review & editing. **Tom van Meerten:** Writing – review & editing. **Thijs H. Oude Munnink:** Writing – review & editing, Visualization, Supervision, Conceptualization. **Bart G.J. Dekkers:** Writing – review & editing, Supervision, Conceptualization.

## Funding

No external funding was received for this research.

## Declaration of competing interest

The authors declare that they have no known competing financial interests or personal relationships that could have appeared to influence the work reported in this paper.

## Data Availability

Data will be made available on request.
