# Supplementary material for: The carbon footprint of Chimeric Antigen Receptor-T cell manufacturing processes
Source: Int J Pharm X. 2026 Aug 28;12:100654. doi: 10.1016/j.ijpx.2026.100654 (PMC13564128; doi:10.1016/j.ijpx.2026.100654)
Supplement: Supplementary file 1 [file mmc1.docx]

**The carbon footprint of Chimeric Antigen Receptor-T cell manufacturing processes**

Wietske E. van der Bijl^1^, Bahez Gareb^1^, Edwin Bremer^2^, Tom van Meerten^2^, Thijs H. Oude Munnink^1,#^, Bart G.J. Dekkers^1,#^

*^1^Department of Clinical Pharmacy and Pharmacology, ^2^Department of Hematology, University of Groningen, University Medical Center Groningen, Groningen, The Netherlands.*

*^#^Shared last authorship.*

**Supplementary Materials**

**Materials and Methods**

## **1. LCA-information**

This study was conducted according to the requirements of ISO 14044:2006.

### **1.1. Goal of the study**

To reduce the impact of CAR-T cell production on the carbon footprint of healthcare, this study aims to determine the carbon emission of different processes in CAR-T cell manufacturing. With that, the processes to reach the CAR-T cell production with the lowest carbon emission can be found. The intended audience is hospitals that are trying to choose between centrally produced and point-of-care CAR-T cells.

### **1.2. Scope of the study**

#### 1.2.1. Function and functional unit

This LCA focusses on the production of CAR-T cells. Although the production processes of axicabtagene ciloleucel (L01XL03) is used for the calculations, the results can be used for all types of CAR-T cells, including axicabtagene ciloleucel (L01XL03), tisagenlecleucel (L01XL04), ciltacabtagene autoleucel (L01XL05), brexucabtagene autoleucel (L01XL06), idecabtagene vicleucel (L01XL07), lisocabtagene maraleucel (L01XL08) and obecabtagene autoleucel (L01XL12). The product system’s function is to produce CAR-T cells, and the functional unit in this study is one individual CAR-T product made for one patient.

#### 1.2.2. System boundary

The system boundary is set at *cradle-to-use.* This is chosen, as the carbon emission of the CAR-T cells is generally similar after use in the end-of-life phase, resulting in no significant change in the overall conclusions of the study. This is in line with the article of Siegert et al (Siegert et al., 2019), that states that a cradle-to-gate analysis should be used when pharmaceutical processes are compared. The flow diagram in figure 1 of the manuscript clearly shows the system boundary of this LCA.

#### 1.2.3. LCIA methodology and types of impacts

Data is either collected from production sites or obtained from other sources. The data is measured, calculated, or estimated. The data in this study is limited to carbon dioxide emissions. The equivalence of the systems being compared is evaluated before interpreting the results.

### **1.3. Life cycle inventory analysis (LCI)**

#### 1.3.1. Collecting data

The data is collected via practical experience (observation of CAR-T production), via protocols or via open access data sources. The exact data sources that are used for the LCI phase can be found in the data collection sheets in supplementary data 2. When no source is given, the data is found in confidential or internal data sources.

#### 1.3.2. Calculating data

Assumptions that were made are stated in supplementary data 2. During the calculation of energy in kWh, kilowatts were multiplied by hours. When amperes were given instead of watts for the energy consumption of an apparatus, a voltage of 230 V and a power factor of 0,8 were assumed (Worldstandards, 2025). With that, equation 1 can be used to calculate the number of watts.

Equation 1: Energy calculation

$$P\left( Watt \right)=I\left( Amperes \right)*V\left( volts \right)*Power factor$$

### **1.4. Life cycle impact assessment (LCIA)**

#### 1.4.1. Impact category and category indicator

The impact category that is being used in this LCA is climate change, which is expressed with the category indicator kg CO_2_e. All LCI results are assigned to the impact category climate change, since this is the only impact category present in this LCA.

#### 1.4.2. Calculation of results

The carbon emission factors that were used in this LCA can be found in supplementary data 2. The impact assessment method that was used was ReCiPe 2016 Midpoint (H). The most carbon emission factors for the calculation of carbon emission per kg material were extracted from CarbonCloud (Carboncloud, n.d.). When the carbon emission of the material was not found on that database, other resources were used. For the carbon emission of energy and transport, the website CO_2_emissiefactoren.nl was used. On this website, a carbon emission of 0,27 kg CO_2_e/kWh was stated, which was used during this LCA.

### **1.5. Life cycle interpretation**

The life cycle interpretation can be found in the results, discussion and conclusion section of the manuscript.

## **2. Data collection sheets**

### **2.1. Datasheet of leukapheresis**

The following datasheet is based on the PoC apheresis manual.

|  | Amount needed | Quantity | Unit | Material | Carbon emission factor (kg CO_2_e /unit) | Carbon emission (kg CO_2_e) |
| --- | --- | --- | --- | --- | --- | --- |
| Spectra Optia Electropheresis System  (STEMart, n.d.) | 5 H | 9.2 | kWh^1^ | Energy | 0.27  (CO₂-emissiefactoren.nl, 2026) | 2.484 |
| Spectra Optia IDL-set | 1 set | N/A | kg | N/A | N/A | N/A |
| Anticoagulant Citrate Dextrose Formula A  (Citralabs, n.d.) | 2 L^2^ | 0.0146 | kg | Citric acid | 10.19  (Carboncloud, n.d.) | 0.148774 |
|  |  | 0.044 | kg | Sodium citrate | 11.04  (Carboncloud, n.d.) | 0.48576 |
|  |  | 0.049 | kg | Dextrose | 1.41  (Carboncloud, n.d.) | 0.06909 |
|  |  | 2 | kg | Water, sterile | 0.575  (Agrawal et al., 2025a) | 1.15 |
| Bloodwarmer  (FutureMed, 2025) | 5 H | 1.8 | kWh^1^ | Energy | 0.27  (CO₂-emissiefactoren.nl, 2026) | 0.486 |
| Astotube tubing  (GBUK, n.d.) | 1 set | 0.243000 | kg | Polyvinyl Chloride | 3.41  (Carboncloud, n.d.) | 0.82863 |
| Infectious waste |  | 2.350653 | kg | Infectious waste | 0.569  (Rizan et al., 2021) | 1.337522 |
| Total |  |  |  |  |  | 6.98978 |

*^1^Based on a voltage of 230 V and a power factor of 0.8. ^2^Based on 10:1 ratio of blood: Anticoagulant Citrate Dextrose Solution A for 20 000 mL of blood.Klik of tik om tekst in te voeren.*

### **2.2. Datasheet of cryopreservation**

The following datasheet is based on practical experience and internal procedures for the PoC production of CAR-T cells.

|  | Amount needed | Quantity | Unit | Material | Carbon emission factor (kg CO_2_e /unit) | Carbon emission (kg CO_2_e) |
| --- | --- | --- | --- | --- | --- | --- |
| Storage of the cryopreserved product | | | | | | |
| Cryofreezer  (Worthington industries, 2018) | 21 days^a^; 40-80% filled, 600 capacity*^b^* | 0.217-0.433 | kWh^c^ | Energy | 0.27  (CO₂-emissiefactoren.nl, 2026) | 0.0585-0.1170 |
|  |  | 0.27786-0.55572 | kg | Liquid nitrogen | 0.06  (Carboncloud, n.d.) | 0.01667-0.03334 |
| Freezing a product (2x) | | | | | | |
| TSCD-II  (UniGreenScheme, n.d.) | 1 hour | 0.07 | kWh | Energy | 0.27  (CO₂-emissiefactoren.nl, 2026) | 0.0189 |
| CompoSeal Universal  (Fresenius Kabi, 2013) | 1 hour | 0.4 | kWh | Energy | 0.27  (CO₂-emissiefactoren.nl, 2026) | 0.108 |
| LAF cabinet  (labwinkel.nl, n.d.) | 1 hour | 0.113 | kWh | Energy | 0.27  (CO₂-emissiefactoren.nl, 2026) | 0.03051 |
| Heidolph polymax 1040  (Heidolph, n.d.) | 10 min | 0.0192 | kWh | Energy | 0.27  (CO₂-emissiefactoren.nl, 2026) | 0.005175 |
| Planer  (CryoProducts, n.d.) | 65 min | 1.3 | kWh^c^ | Energy | 0.27  (CO₂-emissiefactoren.nl, 2026) | 0.351 |
|  | 16L | 12.944 | kg | Liquid nitrogen | 0.06  (Carboncloud, n.d.) | 0.77664 |
| Transferbag 400 mL | 2 | 0.021 | kg | Ethylene vinyl acetate | 2.60857  (ASTM International, 2025) ^f^ | 0.05478 |
| Syringe 50 mL  (SARSTEDT, n.d.) | 1 | 0.01506 | kg^d^ | Polypropylene | 4.77  (Carboncloud, n.d.) | 0.0718362 |
|  |  | 0.01506 | kg^d^ | Polyethylene | 2.88  (Rizan et al., 2021) | 0.0433728 |
|  |  | 0.01506 | kg^d^ | Rubber | 2.54693  (Carboncloud, n.d.) | 0.003209132 |
| Needle 18G | 1 | N/A | kg | N/A | N/A | N/A |
| Transferbag 150 mL | 1 | 0.017 | kg | Ethylene vinyl acetate | 2.60857  (ASTM International, 2025) ^f^ | 0.044345714 |
| Freezing bag 250 | 1 | 0.019 | kg | Ethylene vinyl acetate | 2.60857  (ASTM International, 2025) ^f^ | 0.049562857 |
| DMSO 100% | N/A mL | N/A | kg | DMSO | N/A | N/A |
| Formulation buffer | N/A mL | N/A | kg | N/A | N/A | N/A |
| Infectious waste |  | 0.057 | kg | Infectious waste | 0.569  (Rizan et al., 2021) | 0.032 |
| Liquid nitrogen needed for transport (2x) | | | | | | |
| Liquid nitrogen | 20 L^e^ | 16.18 | kg | Liquid nitrogen | 0.06  (Carboncloud, n.d.) | 0.9708 |
| Thawing (2x) | | | | | | |
| Waterbath  (ThermoFischer, n.d.) | 20 min | 0.2667 | kWh | Energy | 0.27  (CO₂-emissiefactoren.nl, 2026) | 0.072 |
| Total |  |  |  |  |  | 5.34032-5.41549 |

*^a^During centralized production, the time between apheresis and infusion is approximately 4-6 and the production time is 7 days, resulting in a 21-day period of cryopreservation. ^b^Based on local experience, a cryofreezer is usually 40-80% filled, indicating between 214 and 428 IV bags in one cryofreezer. ^c^Based on a voltage of 230V and a power factor of 0.8. ^e^Total weight of the syringe was divided over the three materials. ^e^Based on local experience, 20 L liquid nitrogen is needed for transport from Groningen to Hoofddorp. For international transport, more liquid nitrogen might be needed. ^f^ Based on STRATO EVA film, which is for >98% composed of ethylene vinyl acetate*

### **2.3. Datasheet of transport**

The following datasheet is based on distances and travel times that were determined with Google Maps.

|  | Amount needed | Quantity | Unit | Material | Carbon emission factor (kg CO_2_e/unit) | Carbon emission (kg CO_2_e) |
| --- | --- | --- | --- | --- | --- | --- |
| PoC+  transport | 2x round trip^c^ | 782 | km*^a^* | Diesel B7 | 0.3494825  (CO₂-emissiefactoren.nl, 2026) ^b^ | 273.39517 |
|  |  | 536 | Min^a^ | Transport cooling unit | 0.095  (van Mensch et al., 2024) | 50.92 |
|  |  |  |  | *Total* |  | 324.31517 |
| Centralized transport | 3x round trip^c^ | 625 | km^d^ | Diesel B7 | 0.3494825  (CO₂-emissiefactoren.nl, 2026) ^b^ | 218.37659 |
| Intercontinental transport | 2x round trip | 427 | Km^e^ | Diesel B7 | 0.3494825  (CO₂-emissiefactoren.nl, 2026) ^b^ | 149.05918 |
|  | 1x round trip | 1260 | Ton*km^f^ | Air transport | 0.55  (CO₂-emissiefactoren.nl, 2026) | 693 |
|  |  |  |  | *Total* |  | 842.05918 |
| European transport | 3x round trip^c^ | 5178 | Km^g^ | Diesel B7 | 0.3494825  (CO₂-emissiefactoren.nl, 2026) ^b^ | 1809.62039 |
| European transport, shared | 3x round trip^c^ | 5178 | Km^g^ | Diesel B7 | 0.3494825  (CO₂-emissiefactoren.nl, 2026) ^b^ | 452.57984^h^ |
| European transport, by plane | 2x round trip | 427 | Km^i^ | Diesel B7 | 0.3494825  (CO₂-emissiefactoren.nl, 2026) ^b^ | 60.11099 |
|  | 1x round trip | 1260 | Ton*km^j^ | Air transport | 0.55  (CO₂-emissiefactoren.nl, 2026) | 51.59 |
|  |  |  |  | *Total* |  | 111.70099 |

*^a^ Mean distance/time from all Dutch academic hospitals to the UMCG, multiplied by 4 (2x round trip). ^b^Calculated with a diesel consumption of 10.75 L/100 km (Autoweek, n.d.) (Mercedes Benz Sprinter) and a carbon emission of 3.251 kg CO_2_e/L (CO₂-emissiefactoren.nl, 2026). ^c^ In case of academic transport, the van drives twice between the central production facility and the hospital. Once for picking up the apheresis product and once for returning the CAR-T cells. For central transport, the van drives once more for the delivery of the transport packaging. ^d^Mean distance from all academic hospitals in the Netherlands to KitePharma (Hoofddorp), multiplied by 6 (3x round trip). ^e^Mean distance from all academic hospitals in the Netherlands to Schiphol airport in addition to the distance from LAX airport to KitePharma* (*Santa Monica, USA). ^f^Calculated by multiplying flight distance of 9000 km by the weight of 70 kg by 2 (round trip).* ^g^ *Distance from LMU Klinikum, Germany to KitePharma (Hoofddorp), multiplied by 6 (3x round trip* ^c^*).* ^h^ *Shared transport with 4 CAR-T products per van.* ^i^ *Round trip distance from KitePharma (Hoofddorp) to Schiphol airport in addition to the round trip distance from Munich airport to LMU Klinikum, Germany.* ^j^ *Calculated by multiplying flight distance of 670 km by the weight of 70 kg by 2 (round trip).*

### **2.4. Datasheet of production in open system**

|  | Amount needed | Quantity | Unit | Material | Carbon emission factor (kg CO_2_e/unit) | Carbon emission (kg CO_2_e) |
| --- | --- | --- | --- | --- | --- | --- |
| Spike  (Oncosem, n.d.) | 10 | 0.053 | kg | polypropylene | 4.77  (Carboncloud, n.d.) | 0.25281 |
| clinimacs pbs/edta buffer | 3000 mL | *0.032* | *kg* | *Polypropylene* | 4.77  (Carboncloud, n.d.) | *0.15264* |
|  |  | *0.024* | *kg* | *NaCl* | *0.06*  (Carboncloud, n.d.) | *0.00144* |
|  |  | *0.00057* | *kg* | *KCL* | 0.27  (CO₂-emissiefactoren.nl, 2026) | *0.0001539* |
|  |  | *0.00345* | *kg* | *Na2HPO4* | *N/A* | *N/A* |
|  |  | *0.00057* | *kg* | *KH2PO4* | *0.2649*  *(Edwards et al., 2017)* | *0.000150993* |
|  |  | *0.00111* | *kg* | *Na2EDTA x 2H2O* | *N/A* | *N/A* |
|  |  | *3* | *L* | *H2O (sterile)* | *0.575*  (Agrawal et al., 2025b) | *1.725* |
| Albuman 200 mg/g  (APG Pharma, n.d.; Prothya Biosolutions Netherlands B.V., 2025) | 2x 50 mL | 0.089 | kg | Glass | 1.436967  (Circular ecology, 2025) | 0.127890069 |
|  |  | 7.66667E-05 | kg | Sodium caprylate | *N/A* | *N/A* |
|  |  | 7.66667E-05 | kg | Sodium Chloride | *0.06*  (Carboncloud, n.d.) | 0.0000046 |
|  |  | 7.66667E-05 | kg | Sodium hydroxide | 0.12  (Carboncloud, n.d.) | 0.0000092 |
|  |  | 0.1 | kg | Water (sterile) | 0.575  (Agrawal et al., 2025b) | 0.0575 |
|  |  | 0.02 | kg | Human albumin | *N/A* | *N/A* |
| 50 mL syringe  (Rizan et al., 2023) | 35 | 0.5271 | kg | Polypropylene | 4.77  (Carboncloud, n.d.) | 2.514267 |
|  |  | 0.5271 | kg | polyethylene | 2.88  (Rizan et al., 2021) | 1.518048 |
|  |  | 0.0441 | kg | Rubber | 2.54693  (Circular ecology, 2025) | 0.112319621 |
| IL-7 cytokine | 2 | *N/A* | *N/A* | *N/A* | *N/A* | *N/A* |
| IL-15 cytokine | 2 | *N/A* | *N/A* | *N/A* | *N/A* | *N/A* |
| TEXMACS GMP medium | 2 | 0.08 | kg | Ethylene vinyl acetate | 2.60857^a^  (ASTM International, 2025) | *0.208685714* |
|  |  | 0.08 | kg | Ethylene vinyl alcohol | 2.5  (Carboncloud, n.d.) | *0.2* |
|  |  | 6 | kg | TEXMACS medium | *N/A* | *N/A* |
| 10-15 mL syringe  (Wiedmann et al., 2019) | 15 | 0.12225 | kg | Polypropylene | 4.77  (Carboncloud, n.d.) | 0.5831325 |
|  |  | 0.0102 | kg | Elastomer | 4.94  (Carboncloud, n.d.) | 0.050388 |
|  |  | 0.00396 | kg | Paper | 1.49  (Circular ecology, 2025) | 0.0059004 |
|  |  | 0.0168 | kg | Polyethylene | 2.88  (Rizan et al., 2021) | 0.048384 |
| 400 mL transferbag | 3 | 0.063 | kg | Ethylene vinyl acetate | 2.60857^a^  (ASTM International, 2025) | 0.16434 |
| CD4 reagent | 1 | *N/A* | kg | CD4 microbeads | *N/A* | *N/A* |
|  |  | 0.010866 | kg | borosilicate glass | 2.98  (Wiedmann et al., 2019) | 0.03238068 |
| CD8 reagent | 1 | *N/A* | kg | CD8 microbeads | *N/A* | *N/A* |
|  |  | 0.010866 | kg | borosilicate glass | 2.98  (Wiedmann et al., 2019) | 0.03238068 |
| Column | 1 | *N/A* | *N/A* | *N/A* | *N/A* | *N/A* |
| MidiMACS separator | 1 | *N/A* | *N/A* | *N/A* | *N/A* | *N/A* |
| Culture bag (1L) | 1 | 0.016 | kg | Polypropylene^c^ | 4.77  (Carboncloud, n.d.) | 0.07632 |
|  |  | 0.016 | kg | Polyethylene^c^ | 2.88  (Rizan et al., 2021) | 0.04608 |
| Blood bag centrifuge  (Analis, n.d.) | 4h | 16.8 | kWh | Energy | 0.27  (CO₂-emissiefactoren.nl, 2026) | 4.536 |
| CliniMACS CD4 GMP Microbeads | 1 | *N/A* | *N/A* | *N/A* | *N/A* | *N/A* |
| CliniMACS CD8 GMP Microbeads | 1 | *N/A* | *N/A* | *N/A* | *N/A* | *N/A* |
| T Cell transact | 1 | 0.010866 | kg | borosilicate glass | 2.98  (Wiedmann et al., 2019) | 0.03238068 |
|  |  | *N/A* | kg | rh CD3 and CD28 agonists | *N/A* | *N/A* |
|  |  | 4 | mL | PBS | *N/A* | *N/A* |
|  |  | 0.0012 | mL | Poloxamer 188 | *N/A* | *N/A* |
|  |  | 0.00002 | kg | rh serum albumin | *N/A* | *N/A* |
| Lentiviral vector | 1 | *N/A* | *N/A* | *N/A* | *N/A* | *N/A* |
| Shaker  (Heidolph, n.d.) | 2h | 0.0736 | kWh | Energy | 0.27  (CO₂-emissiefactoren.nl, 2026) | 0.019872 |
| Fomulation solution | 1000 mL | 0.016 | kg | Ethylene vinyl acetate | 2.60857^a^  (ASTM International, 2025) | 0.041737143 |
|  |  | 0.016 | kg | Ethylene vinyl alcohol | 2.5  (Carboncloud, n.d.) | 0.04 |
|  |  | 0.004731 | kg | Sodium chloride | 0.06  (Carboncloud, n.d.) | 0.00028386 |
|  |  | 0.004521 | kg | Sodium gluconate | *N/A* | *N/A* |
|  |  | 0.003308 | kg | sodium acetate trihydrate | 3.81  (Carboncloud, n.d.) | 0.01260348 |
|  |  | 0.002892 | kg | Trisodium citrate dihydrate | 11  (Carboncloud, n.d.) | 0.031812 |
|  |  | 0.000336 | kg | Potassium chloride | 0.27  (CO₂-emissiefactoren.nl, 2026) | 0.00009072 |
|  |  | 0.000275 | kg | Magnesium chloride hexahydrate | *N/A* | *N/A* |
|  |  | 0.0025 | kg | Human albumin | *N/A* | *N/A* |
|  |  | 0.959 | kg | H2O (sterile) | 0.575  (Agrawal et al., 2025b) | 0.551425 |
| 400 mL formulation bag | 1 | 0.021 | kg | Ethylene vinyl acetate | 2.60857 ^a^  (ASTM International, 2025) | 0.05478 |
| Collection bag. 5 L | 1 | 0.05 | kg | Polypropylene^c^ | 4.77  (Carboncloud, n.d.) | 0.2385 |
|  |  | 0.05 | kg | Polyethylene^c^ | 2.88  (Rizan et al., 2021) | 0.144 |
| 1.5 mL Eppendorf  (SARSTEDT, n.d.) | 15 | 0.015 | kg | polypropylene | 4.77  (Carboncloud, n.d.) | 0.07155 |
| Incubator  (Remma, n.d.) | 7d | 13.44 | kWh | Energy | 0.27  (CO₂-emissiefactoren.nl, 2026) | 3.6288 |
| Infectious waste |  | 11.987621 | kg | Infectious waste | 0.569  (Rizan et al., 2021) | 6.820956349 |
| Total |  |  |  |  |  | 24.13502 |

*^a^Based on STRATO EVA film, which is for >98% composed of ethylene vinyl acetate (EVA). ^b^The emission factor is based on the main component. ^c^Polyolefin was undefined, so the following was assumed: 50% polyethylene and 50% polypropylene.*

### **2.5. Datasheet of production in closed system**

The following datasheet is based on the manufacturing protocol of PoC CAR-T cells in the UMCG and practical experience. In the datasheet, the staff is excluded as a factor for carbon emission.

|  | Amount needed | Quantity | Unit | Material | Carbon emission factor (kg CO_2_e/unit) | Carbon emission (kg CO_2_e) |
| --- | --- | --- | --- | --- | --- | --- |
| CliniMACS PBS/EDTA Buffer | 3000 mL | *0.032* | *kg* | *Polypropylene* | 4.77  (Carboncloud, n.d.) | *0.15264* |
|  |  | *0.024* | *kg* | *NaCl* | *0.06*  (Carboncloud, n.d.) | *0.00144* |
|  |  | *0.00057* | *kg* | *KCL* | 0.27  (CO₂-emissiefactoren.nl, 2026) | *0.0001539* |
|  |  | *0.00345* | *kg* | *Na2HPO4* | *N/A* | *N/A* |
|  |  | *0.00057* | *kg* | *KH2PO4* | *0.2649*  *(Edwards et al., 2017)* | *0.000151* |
|  |  | *0.00111* | *kg* | *Na2EDTA x 2H2O* | *N/A* | *N/A* |
|  |  | *3* | *L* | *Water, sterile* | *0.575*  *(Agrawal et al., 2025b)* | *1.725* |
| CliniMACS CD4 GMP Microbeads | 1 | *N/A* | *N/A* | *N/A* | *N/A* | *N/A* |
| CliniMACS CD8 GMP Microbeads | 1 | *N/A* | *N/A* | *N/A* | *N/A* | *N/A* |
| CliniMACS CD4 selection reagent | 1 | *N/A* | kg | CD4 microbeads | *N/A* | *N/A* |
|  |  | 0.010866 | kg | borosilicate glass | 2.98  (Wiedmann et al., 2019) | 0.0323807 |
| CliniMACS CD8 selection reagent | 1 | *N/A* | kg | CD8 microbeads | *N/A* | *N/A* |
|  |  | 0.010866 | kg | borosilicate glass | 2.98  (Wiedmann et al., 2019) | 0.0323807 |
| MACS GMP T Cell TransAct (Reagent) | 1 vial | 0.010866 | kg | borosilicate glass | 2.98  (Wiedmann et al., 2019) | 0.0323807 |
|  |  | *N/A* | kg | recombinant humanized CD3 and CD28 agonists | *N/A* | *N/A* |
|  |  | 4 | mL | Phosphate-Buffered Saline | *N/A* | *N/A* |
|  |  | 0.0012 | mL | Poloxamer 188 | *N/A* | *N/A* |
|  |  | 0.00002 | kg | recombinant Human serum albumin | *N/A* | *N/A* |
| TexMACS GMP Medium | 2 | 0.08 | kg | Ethylene vinyl acetate | 2.60857  (ASTM International, 2025) ^a^ | *0.2086857* |
|  |  | 0.08 | kg | Ethylene vinyl alcohol | 2.5  (Carboncloud, n.d.) | *0.2* |
|  |  | 6 | kg | TEXMACS medium | *N/A* | *N/A* |
| Lentiviral Vector CD19 A3B1-BBZ CAR | 1 vial | *N/A* | *N/A* | *N/A* | *N/A* | *N/A* |
| MACS GMP Recombinant Human IL-7 | 1 vial | *N/A* | *N/A* | *N/A* | *N/A* | *N/A* |
| MACS GMP Recombinant Human IL-15 | 1 vial | *N/A* | *N/A* | *N/A* | *N/A* | *N/A* |
| Albuman 200 mg/g  (APG Pharma, n.d.; Prothya Biosolutions Netherlands B.V., 2025) | 100 mL (over 2 vials) | 0.089 | kg | Glass | 1.436967  (Circular Ecology, 2025) | 0.1278901 |
|  |  | 7.67E-05 | kg | Sodium caprylate | *N/A* | *N/A* |
|  |  | 7.67E-05 | kg | Sodium Chloride | *0.06*  (Carboncloud, n.d.) | 0.0000046 |
|  |  | 7.67E-05 | kg | Sodium hydroxide | 0.12  (Carboncloud, n.d.) | 0.0000092 |
|  |  | 0.1 | kg | Water, sterile | 0.575  (Agrawal et al., 2025b) | 0.0575 |
|  |  | 0.02 | kg | Human albumin | *N/A* | *N/A* |
| CliniMACS Formulation Solution | 1000 mL | 0.016 | kg | Ethylene vinyl acetate | 2.60857  (ASTM International, 2025) ^a^ | 0.0417371 |
|  |  | 0.016 | kg | Ethylene vinyl alcohol | 2.5  (Carboncloud, n.d.) | 0.04 |
|  |  | 0.004731 | kg | Sodium chloride | 0.06  (Carboncloud, n.d.) | 0.0002839 |
|  |  | 0.004521 | kg | Sodium gluconate | *N/A* | *N/A* |
|  |  | 0.003308 | kg | sodium acetate trihydrate | 3.81  (Carboncloud, n.d.) | 0.0126035 |
|  |  | 0.002892 | kg | Trisodium citrate dihydrate | 11  (Carboncloud. n.d.) | 0.031812 |
|  |  | 0.000336 | kg | Potassium chloride | 0,27  (CO₂-emissiefactoren.nl. 2026) | 9.072E-05 |
|  |  | 0.000275 | kg | Magnesium chloride hexahydrate | *N/A* | *N/A* |
|  |  | 0.0025 | kg | Human albumin | *N/A* | *N/A* |
|  |  | 0.959 | kg | Water, sterile | 0.575  (Agrawal et al., 2025b) | 0.551425 |
| CliniMACS Prodigy TS 520 | 1x System tube | 1.14 | kg | Polyvinylchloride | 3.41  (Carboncloud, n.d.) | 3.8874 |
|  | 1x Sample pillow | *N/A* | kg | Polyvinylchloride | 3.41  (Carboncloud, n.d.) | *N/A* |
|  | 1x Pump tube assembly | *N/A* | kg | Silicone (PC) *^b^* | 5  (Carboncloud. n.d.) | *N/A* |
|  | 1x Centrifugation/cultivation chamber | *N/A* | kg | PC, PBT, FKM/FEP, stainless steel, SilCor SiC, PTFE/polyester, MABS, PFPE | *N/A* | *N/A* |
|  | 1x T-connector | *N/A* | kg | Polyvinylchloride | 3.41  (Carboncloud, n.d.) | *N/A* |
|  | 1x 4-way connector | *N/A* | kg | Polyvinylchloride | 3.41  (Carboncloud, n.d.) | *N/A* |
|  | 1x Y-connector | *N/A* | kg | Polyvinylchloride | 3.41  (Carboncloud, n.d.) | *N/A* |
|  | 1x Luer thread (at waste bags) | *N/A* | kg | Polycarbonate | 6  (Boustead, 2005) | *N/A* |
|  | 1x Check valve | *N/A* | kg | methyl methacrylate-acrylonitrile-butadiene-styrene, silicone, styrol-/acrylnitril copolymer | *N/A* | *N/A* |
|  | 1x Drip chamber | *N/A* | kg | Polyvinylchloride | 3.41  (Carboncloud, n.d.) | *N/A* |
|  | 1x Luer locks | *N/A* | kg | methyl methacrylate-acrylonitrile-butadiene-styrene/polycarbonate | *N/A* | *N/A* |
|  | 1x Vented vial adapter | *N/A* | kg | Polycarbonate (PTFE) *^b^* | 6  (Boustead, 2005) | *N/A* |
|  | 1x Non ventilated male luer cap | *N/A* | kg | Polypropylene | 4.77  (Carboncloud, n.d.) | *N/A* |
|  | 1x Tubing set columns | *N/A* | kg | PPA, PP, Fe | *N/A* | *N/A* |
|  | 1x air filter | *N/A* | kg | Polyvinylchloride (PTFE) *^b^* | 3.41  (Carboncloud, n.d.) | *N/A* |
|  | 1x medium filter | *N/A* | kg | modified acrylic, PES, PTFE | *N/A* | *N/A* |
|  | 1x Reagent filter | *N/A* | kg | acrylic resin, PES | *N/A* | *N/A* |
|  | 1x Heat exchange cartridge | *N/A* | kg | polystyrene (AI) *^b^* | 3.43  (Circular ecology, 2025) | *N/A* |
|  |  |  |  |  |  |  |
|  | 1x Spike | *N/A* | kg | Acrylonitrile Butadiene Styrene | 3.125311 | *N/A* |
|  | 2x Waste bags | 0.2 | kg | Polypropylene | 4.77  (Carboncloud, n.d.) | 0.954 |
|  | 4x bags (application bag, target cell bag, reapplicatioon bag, non-target cell bag) | 0.125 | kg | Ethylene vinyl acetate | 2.60857  (ASTM International, 2025) ^a^ | 0.3260714 |
|  | 1x Filters in applicaiton/filtration bag | *N/A* | kg | Polyethylene Terephthalate | 4.48  (Carboncloud, n.d.) | *N/A* |
|  | 1x Spike injection port | *N/A* | kg | Polycarbonate (bromobutyl, chlorobutyl)*^b^* | 6  (Boustead, 2005) | *N/A* |
|  | 1x Tray | *N/A* | kg | Polyethylene Terephthalate | 4.48  (Carboncloud, n.d.) | *N/A* |
|  | 1x Tyvek lid | *N/A* | kg | Tyvek | N/A | *N/A* |
|  | 1x Generic mounting sheet | *N/A* | kg | Polyethylene Terephthalate | 4.48  (Carboncloud, n.d.) | *N/A* |
|  | 1x carrier foil filter | *N/A* | kg | Polyethylene Terephthalate | 4.48  (Carboncloud, n.d.) | *N/A* |
|  | handgrip carrier foils | *N/A* | kg | Polyethylene Terephthalate | 4.48  (Carboncloud, n.d.) | *N/A* |
| CliniMACS Prodigy | 7d | 34.02 | kWh*^c^* | Energy | 0.27  (CO₂-emissiefactoren.nl, 2026) | 9.1854 |
| CliniMACS Prodigy accessoires: Triple Sampling Adapter | 1 | *N/A* | *N/A* | *N/A* | *N/A* | *N/A* |
| CliniMACS Prodigy accessoires: Single Sampling Adapter | 1 | *N/A* | *N/A* | *N/A* | *N/A* | *N/A* |
| Luer/Spike Interconnector | 1 | *N/A* | *N/A* | *N/A* | *N/A* | *N/A* |
| Cytokine vial adapter | 1 | *N/A* | *N/A* | *N/A* | *N/A* | *N/A* |
| Sterile syringe Plastipak 50 mL LL  (Rizan et al., 2023) | 1 | 0.01506 | kg | Polypropylene | 4.77  (Carboncloud, n.d.) | 0.0718362 |
|  |  | 0.01506 | kg | polyethylene | 2.88  (Rizan et al., 2021) | 0.0433728 |
|  |  | 0.00126 | kg | Rubber | 2.54693  (Circular ecology, 2025) | 0.0032091 |
| Collection bag. 5 liters | 1 | 0.05 | kg | polypropylene | 4.77  (Carboncloud, n.d.) | 0.2385 |
|  |  | 0.05 | kg | polyethylene | 2.88  (Rizan et al., 2021) | 0.144 |
| Transfer bags, 150 mL | 1 | 0.017 | kg | Ethylene vinyl acetate | 2.60857  (ASTM International, 2025) ^a^ | 0.0443457 |
| Transfer bags, 400 mL | 1 | 0.021 | kg | Ethylene vinyl acetate | 2.60857  (ASTM International, 2025) ^a^ | 0.05478 |
| Spike  (Oncosem, n.d.) | 1 | 0.0053 | kg | Polypropylene | 4.77  (Carboncloud, n.d.) | 0.025281 |
| Infectious waste |  | 12.10749 | kg | Infectious waste | 0.569  (Rizan et al., 2021) | 6.8891624 |
| Total |  |  |  |  |  | 25.115927 |

*^a^Based on STRATO EVA film, which is for >98% composed of ethylene vinyl acetate (EVA). ^b^The emission factor is based on the main component. ^c^The energy consumption is based on 25% of the maximum energy consumption of 810W during a week.*

### **2.6. Datasheet of background facilities**

The following datasheet is based on one CAR-T product being produced at a time in one cleanroom of 15 m^2^, with personnel and material airlocks of 10 m^2^. In reality, more CAR-T cells can be produced in one cleanroom. For example, multiple bioreactors can be placed in one room, and one LAF cabinet can be used for multiple CAR-T cells. Clothing that was needed inside the cleanrooms was not included in the scope of this study.

|  | Amount needed | Quantity | Unit | Material | Carbon emission factor (kg CO_2_e/unit) | Carbon emission (kg CO_2_e) |
| --- | --- | --- | --- | --- | --- | --- |
| Open system background facilities | | | | | | |
| LAF cabinet (Class A)  (labwinkel.nl, n.d.) | 13 h^b^ | 1.469 | kWh | Energy | 0.27  (CO₂-emissiefactoren.nl, 2026) | 0.39663 |
| Class B  (Bunnak et al., 2016) | 7 d | 245.6712329 | kWh^a^ | Energy | 0.27  (CO₂-emissiefactoren.nl, 2026) | 66.33123 |
| Class C  (Bunnak et al., 2016) | 7 d | 45.45205479 | kWh^a^ | Energy | 0.27  (CO₂-emissiefactoren.nl, 2026) | 12.27205 |
| Class D  (Bunnak et al., 2016) | 7 d | 22.82191781 | kWh^a^ | Energy | 0.27  (CO₂-emissiefactoren.nl, 2026) | 6.16192 |
| Total |  |  |  |  |  | 85.16184 |
| Closed system background facilities | | | | | | |
| LAF cabinet (Class A)  (labwinkel.nl, n.d.) | 1 h | 0.113 | kWh | Energy | 0.27  (CO₂-emissiefactoren.nl, 2026) | 0.03051 |
| Class B  (Bunnak et al., 2016) | 1 h | 1.462328767 | kWh^a^ | Energy | 0.27  (CO₂-emissiefactoren.nl, 2026) | 0.39484 |
| Class C  (Bunnak et al., 2016) | 7 d | 68.17808219 | kWh^a^ | Energy | 0.27  (CO₂-emissiefactoren.nl, 2026) | 18.40808 |
| Class D  (Bunnak et al., 2016) | 7 d | 22.82191781 | kWh^a^ | Energy | 0.27  (CO₂-emissiefactoren.nl, 2026) | 6.16192 |
| Total |  |  |  |  |  | 24.99534 |

^a^*Based on B: 1.52 kW/m^2^, C: 0.5 kW/m^2^, D: 0.41 kW/m^2^ with an area of 15 m^2^ per production area and 10 m^2^ for Personnel and Material airlocks. ^b^Estimation of the time that the LAF cabinet is being used.*

### **2.7. Datasheets of in process controls/quality controls**

In the UMCG, a total of 2 sterility assays, 4 PCR’s, 2 endotoxin tests, 8 flow cytometry and 1 beads-based potency are performed as controls.

|  | Supplementary material No. | Frequency performed | Carbon emission of 1 test (kg CO_2_e) | Total carbon emission of test (kg CO_2_e) |
| --- | --- | --- | --- | --- |
| Sterility | 2.7.1 | 2 | 0.2339 | 0.46785 |
| PCR | 2.7.2 | 4 | 2.117 | 8.46815 |
| Endotoxins | 2.7.3 | 2 | 1.155 | 2.31072 |
| Flow cytometry | 2.7.4 | 8 | 0.163 | 1.30476 |
| Potency | 2.7.5 | 1 | 2.620 | 2.61979 |
| Total |  |  |  | 15.17127 |

#### **2.7.1. Datasheet of sterility assay**

The following datasheet is based on European Pharmacopoeia Chapter 2.6.1, Sterility (European Directorate for the Quality of Medicines & HealthCare (EDQM), 2011a).

|  | Amount needed | Quantity | Unit | Material | Carbon emission factor (kg CO_2_e/unit) | Carbon emission (kg CO_2_e) |
| --- | --- | --- | --- | --- | --- | --- |
| Fluid thioglycollate medium | 0.025 L^a^ | 0.000125 | kg | Pancreatic digest of Casein | 30.1  (Carboncloud, n.d.) | 0.0037625 |
|  |  | 0.06 | kg | Sodium chloride | 0.06  (Carboncloud, n.d.) | 0.00000375 |
|  |  | N/A | kg | Sodium hydroxide 1M | 1.12  (Carboncloud, n.d.) | N/A |
|  |  | 0.025 | mL | Resazurin sodium solution | N/A | N/A |
|  |  | 0.000125 | kg | Glucose | 1.41  (Carboncloud, n.d.) | 0.00017625 |
|  |  | 0.025 | kg | Water R | 0.575  (Agrawal et al., 2025c) | 0.014375 |
|  |  | 0.0000125 | kg | L-cystine | 37.8  (Carboncloud, n.d.) | 0.0004725 |
|  |  | 0.00001875 | kg | Agar | 13.4  (Carboncloud, n.d.) | 0.00025125 |
|  |  | 0.000125 | kg | Yeast extract | 3.34  (Carboncloud, n.d.) | 0.0004175 |
|  |  | 0.0000125 | kg | Sodium thioglycate | N/A | N/A |
| Filter paper | 1 piece | 0.0042 | kg | Mixed paper and board | 1.28850358  (Department for Energy Security and Net Zero, 2025) | 0.00541172 |
| Petri dish | 1 piece | 0.0074 | kg | Polystyrene | 3.43  (Circular ecology, 2025) | 0.025382 |
| Pipette | 1 piece | 0.0104 | kg | Polystyrene | 3.43  (Circular ecology, 2025) | 0.035672 |
| Incubator  (Remma, n.d.) | 14 d | 0.448 | kWh^b^ | Energy | 0.27  (CO₂-emissiefactoren.nl, 2026) | 0.12096 |
| Infectious waste |  | 0.0474813 | kg | Infectious waste | 0.569  (Rizan et al., 2021) | 0.027016831 |
| Total |  |  |  |  |  | 0.23393 |

^a^*Volume of a petri dish.^b^Based on experience, it is estimated that 60 dishes are inside the incubator. The amount of kWh given is already divided by this number.*

#### **2.7.2. Datasheet of PCR**

The following datasheet is based on internal procedures.

|  | Amount needed | Quantity | Unit | Material | Carbon emission factor (kg CO_2_e/unit) | Carbon emission (kg CO_2_e ) |
| --- | --- | --- | --- | --- | --- | --- |
| DNA isolation |  |  |  |  |  |  |
| Promega DNA isolation kit | 1 | N/A | N/A | N/A | N/A | N/A |
| Maxwell RSC cartridge | 10 | N/A | N/A | N/A | N/A | N/A |
| RSC plunger | 10 | N/A | N/A | N/A | N/A | N/A |
| Eppendorf tubes (small)  (SARSTEDT, n.d.) | 10 | 0.002 | kg | Polypropylene | 4.77  (Carboncloud, n.d.) | 0.00954 |
| Elution buffer | 1500 uL | N/A | N/A | N/A | N/A | N/A |
| 200 μL pipette tips  (SARSTEDT, n.d.) | 20^a^ | 0.006 | kg | Polypropylene | 4.77  (Carboncloud, n.d.) | 0.02862 |
| Maxwell CSC  (Promega, n.d.) | 50 min | 0.383 | kWh^b^ | Energy | 0.27  (CO₂-emissiefactoren.nl, 2026) | 0.1035 |
| PCR |  |  |  |  |  |  |
| Vortex | 10 min ^a^ | 0.0033333 | kWh | Energy | 0.27  (CO₂-emissiefactoren.nl, 2026) | 0.0009 |
| Centrifuge | 10 min ^a^ | 0.0533333 | kWh | Energy | 0.27  (CO₂-emissiefactoren.nl, 2026) | 0.0144 |
| Quantstudio 5 | 1.5 h^c^ | 2.346 | kWh | Energy | 0.27  (CO₂-emissiefactoren.nl, 2026) | 0.63342 |
| Plate spinner | 1 min | 0.0041667 | kWh | Energy | 0.27  (CO₂-emissiefactoren.nl, 2026) | 0.001125 |
| PCR hood | 2 h ^a^ | 0.128 | kWh | Energy | 0.27  (CO₂-emissiefactoren.nl, 2026) | 0.03456 |
| Cell counter | 10 min | 0.0313333 | kWh | Energy | 0.27  (CO₂-emissiefactoren.nl, 2026) | 0.00846 |
| 1000 μL pipette tips | 10 tips ^a^ | 0.012 | kg | Polypropylene | 4.77  (Carboncloud, n.d.) | 0.05724 |
| 200 μL pipette tips | 20 tips ^a^ | 0.006 | kg | Polypropylene | 4.77  (Carboncloud, n.d.) | 0.02862 |
| 20 μL pipette tips | 100 tips ^a^ | 0.02 | kg | Polypropylene | 4.77  (Carboncloud, n.d.) | 0.0954 |
| 1.5 mL tubes | 30 tubes ^a^ | 0.03 | kg | Polypropylene | 4.77  (Carboncloud, n.d.) | 0.1431 |
| 96 well plates | 1 plate | 0.169 | kg | Polypropylene | 4.77  (Carboncloud, n.d.) | 0.80613 |
| Adhesive seal for PCR plates | 1 seal | 0.002 | kg | Plastic film | 5.74  (Carboncloud, n.d.) | 0.01148 |
| Taqman Fast Advanced Master Mix | 1000 uL ^a^ | N/A | N/A | N/A | N/A | N/A |
| Forward Primers | 10 uL | N/A | N/A | N/A | N/A | N/A |
| Reverse Primers | 10 uL | N/A | N/A | N/A | N/A | N/A |
| Probes | 10 uL | N/A | N/A | N/A | N/A | N/A |
| gBlocks Gene Fragments | 3.3 uL | N/A | N/A | N/A | N/A | N/A |
| Nucleasefree water | 3 mL ^a^ | N/A | N/A | N/A | N/A | N/A |
| Infectious waste |  | 0.247 | kg | Infectious waste | 0.569  (Rizan et al., 2021) | 0.140543 |
| Total |  |  |  |  |  | 2.11704 |

^a^ *Estimation, based on own experience. ^b^Based on a voltage of 230V and a power factor of 0.8. ^c^Mean of mycoplasma (1h45min) and vector copy number/replicate competent lentivirus (1h10min)*

#### **2.7.3. Datasheet of endotoxins**

The following datasheet is based on European Pharmacopoeia Chapter 2.6.14, Bacterial endotoxins (European Directorate for the Quality of Medicines & HealthCare (EDQM), 2011b).

|  | Amount needed | Quantity | Unit | Material | Carbon emission factor (kg CO_2_e/unit) | Carbon emission (kg CO_2_e) |
| --- | --- | --- | --- | --- | --- | --- |
| 1000 μL pipette tips  (SARSTEDT, n.d.) | 10 tips^a^ | 0.012 | kg | Polypropylene | 4.77  (Carboncloud, n.d.) | 0.05724 |
| 200 μL pipette tips  (SARSTEDT, n.d.) | 30 tips^a^ | 0.009 | kg | Polypropylene | 4.77  (Carboncloud, n.d.) | 0.04293 |
| 20 μL pipette tips  (SARSTEDT, n.d.) | 10 tips^a^ | 0.002 | kg | Polypropylene | 4.77  (Carboncloud, n.d.) | 0.00954 |
| LAL reagent | 2 mL^a^ | N/A | kg | N/A | N/A | N/A |
| Standard endotoxin solution | 50 uL^a^ | N/A | kg | N/A | N/A | N/A |
| Water (sterile) | 20 mL^a^ | [0.02](https://www.sciencedirect.com/science/article/pii/S2772572325000123?utm) | kg | Water, sterile | 0.575  (Agrawal et al., 2025a) | 0.0115 |
| 96-well plate  (SARSTEDT, n.d.) | 1 | 0.169 | kg | Polypropylene | 4.77  (Carboncloud, n.d.) | 0.80613 |
| 1.5 mL tubes  (SARSTEDT, n.d.) | 10 tubes^a^ | 0.01 | kg | Polypropylene | 4.77  (Carboncloud, n.d.) | 0.0477 |
| Incubator  (Remma, n.d.) | 1.5 hours | 0.12 | kWh | Energy | 0.27  (CO₂-emissiefactoren.nl, 2026) | 0.0324 |
| Fluorescence microplate reader  (BioTek, 2008) | 1 hour | 0.08 | kWh^b^ | Energy | 0.27  (CO₂-emissiefactoren.nl, 2026) | 0.0216 |
| Infectious waste |  | 0.222 | kg | Infectious waste | 0.569  (Rizan et al., 2021) | 0.126318 |
| Total |  |  |  |  |  | 1.15536 |

^a^ *Estimation, based on own experience. ^b^Based on a power factor of 0.8.*

#### **2.7.4. Datasheet of flow cytometry**

The following datasheet is based on internal procedures.

|  | Amount needed | Quantity | Unit | Material | Carbon emission factor (kg CO_2_e/unit) | Carbon emission (kg CO_2_e) |
| --- | --- | --- | --- | --- | --- | --- |
| MACSQuant analyzer 10  (Miltenyi Biotec, n.d.) | 5 min | 0.0375 | kWh | Energy | 0.27  (CO₂-emissiefactoren.nl, 2026) | 0.010125 |
| Vortex  (Ltd, n.d.) | 0.5 min^a^ | 0.0001667 | kWh | Energy | 0.27  (CO₂-emissiefactoren.nl, 2026) | 0.000045 |
| Centrifuge  (MPW-351E Laboratory Centrifuge, n.d.) | 15 min^a^ | 0.08 | kWh | Energy | 0.27  (CO₂-emissiefactoren.nl, 2026) | 0.0216 |
| 1000 μL pipette tips  (SARSTEDT, n.d.) | 5 tips^a^ | 0.006 | kg | Polypropylene | 4.77  (Carboncloud, n.d.) | 0.02862 |
| 200 μL pipette tips  (SARSTEDT, n.d.) | 3 tips^a^ | 0.0009 | kg | Polypropylene | 4.77  (Carboncloud, n.d.) | 0.004293 |
| 20 μL pipette tips  (SARSTEDT, n.d.) | 10 tips^a^ | 0.002 | kg | Polypropylene | 4.77  (Carboncloud, n.d.) | 0.00954 |
| 12x75 mm FACS tubes  (SARSTEDT, n.d.) | 1 tube | 0.0028 | kg | Polystyrene | 3.43  (Circular ecology, 2025) | 0.009604 |
| 15 mL conical tubes  (SARSTEDT, n.d.) | 2 tubes^a^ | 0.0126 | kg | Polypropylene | 4.77  (Carboncloud, n.d.) | 0.060102 |
| 1.5 mL tubes  (SARSTEDT, n.d.) | 1 tube | 0.001 | kg | Polypropylene | 4.77  (Carboncloud, n.d.) | 0.00477 |
| Infectious waste |  | 0.0253 | kg | Infectious waste | 0.569  (Rizan et al., 2021) | 0.0143957 |
| Total |  |  |  |  |  | 0.16309 |

^a^*Estimation based on the mean value of protocols for transduction frequency and immune cell composition.*

#### **2.7.5. Datasheet of potency**

The following datasheet is based on internal procedures.

|  | Amount needed | Quantity | Unit | Material | Carbon emission factor (kg CO_2_e /unit) | Carbon emission (kg CO_2_e) |
| --- | --- | --- | --- | --- | --- | --- |
| Cell based | | | | | | |
| Waterbath (37°C) | 1 hour | 0.8 | kWh | Energy | 0.27  (CO₂-emissiefactoren.nl, 2026) | 0.216 |
| MACSQuant Analyzer 10 | 25 min | 0.1875 | kWh | Energy | 0.27  (CO₂-emissiefactoren.nl, 2026) | 0.050625 |
| CO_2_ -incubator | 24 hours | 1.92 | kWh | Energy | 0.27  (CO₂-emissiefactoren.nl, 2026) | 0.5184 |
| LAF-cabinet | 2 hours | 0.226 | kWh | Energy | 0.27  (CO₂-emissiefactoren.nl, 2026) | 0.06102 |
| 5000 μL pipette tips | 3 tips^a^ | 0.0078 | kg | Polypropylene | 4.77  (Carboncloud, n.d.) | 0.037206 |
| 1000 μL pipette tips | 4 tips^a^ | 0.0048 | kg | Polypropylene | 4.77  (Carboncloud, n.d.) | 0.022896 |
| 200 μL pipette tips | 200 Tips^a^ | 0.06 | kg | Polypropylene | 4.77  (Carboncloud, n.d.) | 0.2862 |
| Vortex | 3 min^a^ | 0.001 | kWh | Energy | 0.27  (CO₂-emissiefactoren.nl, 2026) | 0.00027 |
| Shaker | 3 hours | 0.1104 | kWh^b^ | Energy | 0.27  (CO₂-emissiefactoren.nl, 2026) | 0.029808 |
| Centrifuge | 15 min | 0.08 | kWh | Energy | 0.27  (CO₂-emissiefactoren.nl, 2026) | 0.0216 |
| MACSPlex detection reagent | 80 uL | N/A | N/A | N/A | N/A | N/A |
| MACSPlex cytokine capture beads | 80 uL | N/A | N/A | N/A | N/A | N/A |
| MACSPlex buffer | 24 mL^a^ | N/A | N/A | N/A | N/A | N/A |
| Cell lines: NALM6, CEMEV, CEMCD19 | 300 000 cells | N/A | N/A | N/A | N/A | N/A |
| 1.5 mL tubes | 30 tubes | 0.03 | kg | Polypropylene | 4.77  (Carboncloud, n.d.) | 0.1431 |
| 96 well plate | 1 | 0.169 | kg | Polypropylene | 4.77  (Carboncloud, n.d.) | 0.80613 |
| MACSPlex filter plate | 1 | 0.0568 | kg | Polystyrene | 3.43  (Circular ecology, 2025) | 0.194824 |
| T75 culture flask | 1 flask | 0.0502 | kg | Polystyrene | 3.43  (Circular ecology, 2025) | 0.172186 |
| RPMI medium | 4 mL^a^ | N/A | N/A | N/A | N/A | N/A |
| Texmacs | 10 mL | N/A | N/A | N/A | N/A | N/A |
| FCS | 0.4 mL^a^ | N/A | N/A | N/A | N/A | N/A |
| IL7 | N/A | N/A | N/A | N/A | N/A | N/A |
| IL15 | N/A | N/A | N/A | N/A | N/A | N/A |
| Infectious waste |  | 0.3786 | kg | Infectious waste | 0.569  (Rizan et al., 2021) | 0.2154234 |
| Total |  |  |  |  |  | 2.77569 |
| Beads based | | | | | | |
| Ella | 90 min | 0.36 | kWh^b^ | Energy | 0.27  (CO₂-emissiefactoren.nl, 2026) | 0.0972 |
| MACSQuant Analyzer 10 | 25 min | 0.1875 | kWh | Energy | 0.27  (CO₂-emissiefactoren.nl, 2026) | 0.050625 |
| CO2 incubator | 24 hours | 1.92 | kWh | Energy | 0.27  (CO₂-emissiefactoren.nl, 2026) | 0.5184 |
| LAF-cabinet | 2 hours | 0.226 | kWh | Energy | 0.27  (CO₂-emissiefactoren.nl, 2026) | 0.06102 |
| Vacuum |  |  |  |  |  |  |
| 5000 μL pipette tips | 1 tip^a^ | 0.0026 | kg | Polypropylene | 4.77  (Carboncloud, n.d.) | 0.012402 |
| 1000 μL pipette tips | 2 tips^a^ | 0.0024 | kg | Polypropylene | 4.77  (Carboncloud, n.d.) | 0.011448 |
| 200 μL pipette tips | 180 Tips^a^ | 0.054 | kg | Polypropylene | 4.77  (Carboncloud, n.d.) | 0.25758 |
| 20 μL pipette tips | 30 tips^a^ | 0.006 | kg | Polypropylene | 4.77  (Carboncloud, n.d.) | 0.02862 |
| Vortex | 3 min^a^ | 0.001 | kWh | Energy | 0.27  (CO₂-emissiefactoren.nl, 2026) | 0.00027 |
| Shaker | 3 hours | 0.1104 | kWh^b^ | Energy | 0.27  (CO₂-emissiefactoren.nl, 2026) | 0.029808 |
| Centrifuge | 10 min | 0.0533333 | kWh | Energy | 0.27  (CO₂-emissiefactoren.nl, 2026) | 0.0144 |
| Ella cartridge | 1 | N/A | N/A | N/A | N/A | N/A |
| 96 well plate | 1 | 0.169 | kg | Polypropylene | 4.77  (Carboncloud, n.d.) | 0.80613 |
| MACSPlex filter plate | 1 | 0.0568 | kg | Polystyrene | 3.43  (Circular ecology, 2025) | 0.194824 |
| MACSPlex detection reagent | 80 uL | N/A | N/A | N/A | N/A | N/A |
| MACSPlex cytokine capture beads | 80 uL | N/A | N/A | N/A | N/A | N/A |
| MACSPlex buffer | 17 mL^a^ | N/A | N/A | N/A | N/A | N/A |
| 1.5 mL tubes | 32 tubes^a^ | 0.032 | kg | Polypropylene | 4.77  (Carboncloud, n.d.) | 0.15264 |
| T75 culture flask | 1 flask | 0.0502 | kg | Polystyrene | 3.43  (Circular ecology, 2025) | 0.172186 |
| FCS | 0.2 mL^a^ | N/A | N/A | N/A | N/A | N/A |
| RPMI medium | 2 mL* | N/A | N/A | N/A | N/A | N/A |
| Texmacs | N/A | N/A | N/A | N/A | N/A | N/A |
| IL7 | N/A | N/A | N/A | N/A | N/A | N/A |
| IL15 | N/A | N/A | N/A | N/A | N/A | N/A |
| Beads | 6 000 000 beads | N/A | N/A | N/A | N/A | N/A |
| CD19 CAR Detection Reagent | N/A | N/A | N/A | N/A | N/A | N/A |
| PBS | 2 mL^a^ | N/A | N/A | N/A | N/A | N/A |
| Infectious waste |  | 0.373 | kg | Infectious waste | 0.569  (Rizan et al., 2021) | 0.212237 |
| Total |  |  |  |  |  | 2.61979 |

^a^*Estimation based on practitcal experience and SOP. ^b^Used 0.8 as power factor.*

### **2.8. Datasheet of infusion**

The following datasheet is based on the infusion of 1 product with an IV administration set, that is afterwards being flushed with 0.9% NaCl. Clothing was not included, similar to the background facilities (paragraph 2.6.).

|  | Amount needed | Quantity | Unit | Material | Carbon emission factor (kg CO_2_e /unit) | Carbon emission (kg CO_2_e) |
| --- | --- | --- | --- | --- | --- | --- |
| IV administration set  (Zietse et al., 2025) | 1 set | 0.050 | kg | Polyvinyl Chloride | 3.41  (Carboncloud, n.d.) | 0.1705 |
| 0.9% NaCl | 250 mL | 0.250 | kg | Water, sterile | 0.575  (Agrawal et al., 2025a) | 0.000135 |
|  |  | 0.00225 | kg | Sodium chloride | 0.06  (Carboncloud, n.d.) | 0.14375 |
|  |  | 1 | bag | Infusion bag 250 mL | 0.569  (Drew and Rizan, 2022) | 0.26 |
| Total |  |  |  |  |  | 0.574 |

## **3. Equations**

### **3.1. Cryopreservation**

Equation 1: Calculation of the CO_2_-emission of cryopreservation.

$${\mathrm{CO}_{2}e}_{cryo}=d*\frac{1,5321}{n_{IV-bags}}+n_{freeze-thaw}*0,661765+n_{cryotransport}*{LN}_{transport}*0,04854$$

*CO_2_e_cryo_ = CO_2_-emission of cryopreservation (kg CO_2_e)
d = Time of cryostorage (days)*

*n_IV-bags_= Number of IV bags in the cryofreezer during storage.*

*n_freeze-thaw_= Number of freeze-thaw cycli*

*N_cryotransport_ = Number of cryotransports*

*LN_transport_= Liquid nitrogen needed for transport (L)*

### **3.2. Transport**

Equation 2: Calculation of the CO_2_-emission of cooled transport

$${\mathrm{CO}_{2}e}_{transport, cooled}={km}_{van}*0,3494825+h*5,7$$

CO_2_*e_transport, cooled_ = CO_2_-emission of cooled transport (kg CO_2_e)*

*km_van_ = distance of van transport (km)*

*h= travel time (h)*

Equation 3: Calculation of the CO_2_-emission of (air) transport

$${\mathrm{CO}_{2}e}_{transport}={km}_{van}*0,3494825+{km}_{air}*0,0385$$

CO_2_*e_transport_ = CO_2_-emission of transport (kg CO_2_e)*

*km_van_ = distance of van transport (km)*

*km_air_ = distance of air transport (km)*

### **3.3. Background facilities**

Equation 4: Calculation of the CO_2_-emission of the background facilities

$${\mathrm{CO}_{2}e}_{background}=\frac{t_{p}*(0,02632192*A_{b}+0,007304795*A_{c}+0,003667808*A_{d})}{n}+t_{LAF}*0,03051$$

$\mathrm{CO}_{2}$*e_background_= CO_2_-emission of background facilities (kg CO_2_e)*

*t_p_= Production time (hours)*

*A_b_= Area of cleanroom class B (m^2^)*

*A_c_= Area of cleanroom class C (m^2^)*

*A_d_= Area of cleanroom class D (m^2^)*

*n= number of CAR-T cell productions taking place in the facility*

*t_LAF_= time in the LAF cabinet, class A (hours)*

### **3.4. Quality control**

Equation 5: Calculation of the CO_2_-emission of the sterility assay

$${\mathrm{CO}_{2}e}_{Steility}=\frac{7,2576}{n}+0,112941$$

$\mathrm{CO}_{2}$*e_sterility_= CO_2_-emission of sterility assay (kg CO_2_e)*

*n= number of petri dishes in the incubator during the assay*

**References**

Agrawal, D., Crockett, S., Palchaudhuri, S., Hernandez, L., Skole, K., Shimpi, R., Collins, J., Von Renteln, D., Pohl, H., 2025a. Ripple Effect: Safety, Cost, and Environmental Concerns of Using Sterile Water in Endoscopy. Gastro Hep Advances 4, 100625. https://doi.org/10.1016/j.gastha.2025.100625

Agrawal, D., Crockett, S., Palchaudhuri, S., Hernandez, L., Skole, K., Shimpi, R., Collins, J., Von Renteln, D., Pohl, H., 2025b. Ripple Effect: Safety, Cost, and Environmental Concerns of Using Sterile Water in Endoscopy. Gastro Hep Advances 4, 100625. https://doi.org/10.1016/j.gastha.2025.100625

Agrawal, D., Crockett, S., Palchaudhuri, S., Hernandez, L., Skole, K., Shimpi, R., Collins, J., Von Renteln, D., Pohl, H., 2025c. Ripple Effect: Safety, Cost, and Environmental Concerns of Using Sterile Water in Endoscopy. Gastro hep advances 4, 100625. https://doi.org/10.1016/j.gastha.2025.100625

Analis, n.d. Sigma 8KBS, MD, package, floorstanding refrigerated centrifuge, aircooled compressor, adapters for quadruple or single blood bag systems (SIG-100010) [WWW Document]. URL https://www.analis.com/nl-be/shop/sig-100010-sigma-8kbs-md-package-floorstanding-refrigerated-centrifuge-aircooled-compressor-adapters-for-quadruple-or-single-blood-bag-systems-106703?category=508 (accessed 2.19.26).

APG Pharma, n.d. 100 mL injectieflacon, helder, type 2 geblazen glas [WWW Document]. URL https://apg-pharma.nl/100-ml-injectieflacon-helder-type-2-geblazen-glas-apg-art-nr-1007373 (accessed 2.17.26).

ASTM International, 2025. Environmental Product Declaration (EPD) for Satinal S.P.A. STRATO® EVA Film (EPD 949) [WWW Document]. URL https://www.astm.org/v3/assets/blt5eb0a2cb04534832/blt63abc9ec1c42b3d5/67d87e11ef4ce67a77d27687/Satinal_EPD_for_STRATO_EVA_Film_FINALf_protected.pdf (accessed 2.9.26).

Autoweek, n.d. Verbruik Mercedes Benz Sprinter [WWW Document]. URL https://www.autoweek.nl/verbruiksmonitor/mercedes-benz/sprinter/ (accessed 2.9.26).

BioTek, 2008. Synergy HT Multi-Mode Microplate Reader Operator’s Manual, Revision I [WWW Document]. URL https://www.manualslib.com/manual/1416962/Biotek-Synergy-Ht.html?page=3#manual (accessed 2.10.26).

Boustead, I., 2005. Eco-profiles of the European Plastics Industry - Polycarbonate. Brussels.

Bunnak, P., Allmendinger, R., Ramasamy, S. V., Lettieri, P., Titchener‐Hooker, N.J., 2016. Life‐cycle and cost of goods assessment of fed‐batch and perfusion‐based manufacturing processes for mAbs. Biotechnol. Prog. 32, 1324–1335. https://doi.org/10.1002/btpr.2323

Carboncloud, n.d. Carboncloud — Sustainability intelligence platform for emissions and climate footprint calculations. [WWW Document]. URL https://carboncloud.com/ (accessed 2.9.26).

Circular ecology, 2025. ‘The Inventory of Carbon and Energy (ICE) database’ [WWW Document]. URL https://www.climatiq.io/ (accessed 2.9.26).

Circular Ecology, 2025. The Inventory of Carbon and Energy (ICE) database.

Citralabs, n.d. Anticoagulant citrate dextrose A - anhydrous citric acid, dextrose monohydrate, ant trisodium citrate dihydrate solution. Solution A, U.S.P., PN 9350, 30 mL, NDC 23732-6051-3. .

CO₂-emissiefactoren.nl, 2026. CO₂-emissiefactoren — Reliable CO₂ emission factors for the Dutch market [WWW Document]. URL https://co2emissiefactoren.nl/ (accessed 2.9.26).

CryoProducts, n.d. Planer Kryo 560-16 [WWW Document]. URL https://cryo-products.com/wp-content/uploads/2018/05/CryoProducts_PlanerKryo560.pdf (accessed 2.9.26).

Department for Energy Security and Net Zero, 2025. Greenhouse gas reporting: conversion factors 2025 [WWW Document]. URL https://www.gov.uk/government/publications/greenhouse-gas-reporting-conversion-factors-2025 (accessed 2.9.26).

Drew, J., Rizan, C., 2022. HealthcareLCA Database. HealthcareLCA. [WWW Document]. URL healthcarelca.com/database (accessed 2.9.26).

Edwards, R., Padella, M., Giuntoli, J., Koeble, R., O’Connell, A., Bulgheroni, C., Marelli, L., 2017. Definition of input data to assess GHG default emissions from biofuels in EU legislation, Version 1c. Publications Office of the European Union. https://doi.org/10.2790/658143

European Directorate for the Quality of Medicines & HealthCare (EDQM), 2011a. European Pharmacopoeia (Ph. Eur.), General Chapter 2.6.1: Sterility (Version 04/2011:20601).

European Directorate for the Quality of Medicines & HealthCare (EDQM), 2011b. European Pharmacopoeia (Ph. Eur.), General Chapter 2.6.14: Bacterial endotoxins (Version 01/2018:20614).

Fresenius Kabi, 2013. CompoSeal Universal Operating Instructions [WWW Document]. URL https://www.manualslib.com/manual/2230158/Fresenius-Kabi-Composeal-Universal.html?page=27#manual (accessed 2.9.26).

FutureMed, 2025. AP220 Product Specifications [WWW Document]. URL https://futuremed.com/wp-content/uploads/2025/09/AP220-SPECS.pdf (accessed 2.9.26).

GBUK, n.d. Astotube [WWW Document]. URL https://futuremed.com/wp-content/uploads/2025/09/AP220-SPECS.pdf (accessed 2.9.26).

Heidolph, n.d. Heidolph polymax 1040 wip wap schudder met hoek van 5° (L1298543-42205-00) [WWW Document]. URL https://www.laboratorium-apparatuur.nl/heidolph-polymax-1040-wip-wap-schudder-met-hoek-van-5-l1298543-42205-00 (accessed 2.9.26).

labwinkel.nl, n.d. Labogene Scanlaf Mars 1500 (SCAN-003) [WWW Document]. URL https://www.labwinkel.nl/mars-1500-labogene-scanlaf-mars-1500 (accessed 2.9.26).

Ltd, L.E., n.d. Vortex Mixer LVOM-A17 | Digital Vortex Mixer Supplier | Labtron. Labtron Equipment Ltd - Laboratory Equipment | Analytical Equipment [WWW Document]. URL https://www.labtron.com/vortex-mixers/lvom-a17 (accessed 2.9.26).

Miltenyi Biotec, n.d. MACSQuant Analyzer 10 Flow Cytometer - specifications [WWW Document]. URL https://www.miltenyibiotec.com/UN-en/products/macsquant-analyzer-10.html#full-width-headline-PGgyPkZlYXR1cmVzICYgc3BlY2lmaWNhdGlvbnM8L2gyPg== (accessed 2.10.26).

MPW-351E Laboratory Centrifuge, 2.925, 00€., n.d. ProfiLab24 MPW-351e Laboratory Centrifuge.

Oncosem, n.d. Bag Spike With Negative Needlefree Injection Valve. BS001-NG [WWW Document]. URL https://oncosem.com/en/product/bag-spike-with-negative-needlefree-injection-valve/ (accessed 2.17.26).

Promega, n.d. Maxwell CSC Instrument Operating Manual (Technical manual), Mod. No. 9800-000, Cat. No. AS4000 [WWW Document]. URL https://www.promega.com/-/media/files/resources/protocols/technical-manuals/101/maxwell-csc-instrument-operating-manual.pdf (accessed 2.9.26).

Prothya Biosolutions Netherlands B.V., 2025. Albuman 200 g/l SmPC NL - Samenvatting van de productkenmerken.

Remma, n.d. CO2 incubator HeraCell 150 - Heraeus [WWW Document]. URL https://remma.fr/en/model/co2-incubator-heracell-150 (accessed 2.9.26).

Rizan, C., Bhutta, M.F., Reed, M., Lillywhite, R., 2021. The carbon footprint of waste streams in a UK hospital. J. Clean. Prod. 286, 125446. https://doi.org/10.1016/j.jclepro.2020.125446

Rizan, C., Lillywhite, R., Reed, M., Bhutta, M.F., 2023. The carbon footprint of products used in five common surgical operations: identifying contributing products and processes. J. R. Soc. Med. 116, 199–213. https://doi.org/10.1177/01410768231166135

SARSTEDT, n.d. Sarstedt products specifications [WWW Document]. URL https://www.sarstedt.com/en/products/ (accessed 2.9.26).

Siegert, M.-W., Lehmann, A., Emara, Y., Finkbeiner, M., 2019. Harmonized rules for future LCAs on pharmaceutical products and processes. Int. J. Life Cycle Assess. 24, 1040–1057. https://doi.org/10.1007/s11367-018-1549-2

STEMart, n.d. Terumo Spectra Optia Apheresis System, 100-240 VAC, 10 Amps (Terumo 161627). [WWW Document]. https://www.ste-mart.com/terumo-spectra-optia-apheresis-system-100-240vac-10-amps-terumo-161627.htm?utm. URL https://www.ste-mart.com/terumo-spectra-optia-apheresis-system-100-240vac-10-amps-terumo-161627.htm?utm (accessed 2.9.26).

ThermoFischer, n.d. Product specifications Precision General Purpose Baths, Cat. No. TSGP10. [WWW Document]. URL https://www.thermofisher.com/order/catalog/product/TSGP10/tabs?defaultTab=0 (accessed 2.9.26).

UniGreenScheme, n.d. Terumo TSCD-II Type Terumo Sterile Tubing Welder Lab (ITEM-47119) [WWW Document]. URL https://shop.unigreenscheme.co.uk/laboratory-equipment/plasma-technologies/terumo-tscd-ii-type-terumo-sterile-tubing-welder-lab (accessed 2.9.26).

van Mensch, P., Elstgesst, M., Spreen, J., 2024. Real-world emissions of temperature-controlled road transport in the Netherlands, TNO2024R11647.

Wiedmann, T., Teh, S.H., Yu, M., 2019. ICM Database - Integrated Carbon Metrics Embodied Carbon Life Cycle Inventory Database [WWW Document]. URL http://hdl.handle.net/1959.4/resource/collection/resdatac_762/1 (accessed 2.9.26).

Worldstandards, 2025. Overzichtstabel met stekkers, netspanning & frequentie per land [WWW Document]. URL https://www.worldstandards.eu/nl/elektriciteit/landenoverzicht-stekkers-voltage/ (accessed 2.17.26).

Worthington industries, 2018. Instruction Manual - Medical Device K SERIES Cryostorage System [WWW Document]. URL https://www.manualslib.com/manual/1947173/Worthington-K-Series.html#manual (accessed 2.12.26).

Zietse, M., Kooijman, T., Malmberg, R., Spierings, L.E.A.M.M., Jager, A., Mathijssen, R.H.J., van Leeuwen, R.W.F., Thielen, F.W., 2025. Environmental Impact Assessment of Intravenous Versus Subcutaneous Monoclonal Antibodies: A Carbon Footprint Analysis. JCO Oncol. Pract. 21, 1287–1295. https://doi.org/10.1200/OP-24-00804
